# The Sweet Potato NAC-Domain Transcription Factor IbNAC1 Is Dynamically Coordinated by the Activator IbbHLH3 and the Repressor IbbHLH4 to Reprogram the Defense Mechanism against Wounding

**DOI:** 10.1371/journal.pgen.1006397

**Published:** 2016-10-25

**Authors:** Shi-Peng Chen, Chih-Hsien Kuo, Hsueh-Han Lu, Hui-Shan Lo, Kai-Wun Yeh

**Affiliations:** Institute of Plant Biology, National Taiwan University, Taipei, Taiwan; University of California Davis, UNITED STATES

## Abstract

IbNAC1 is known to activate the defense system by reprogramming a genetic network against herbivory in sweet potato. This regulatory activity elevates plant defense potential but relatively weakens plants by IbNAC1-mediated JA response. The mechanism controlling *IbNAC1* expression to balance plant vitality and survival remains unclear. In this study, a wound-responsive G-box *cis*-element in the *IbNAC1* promoter from -1484 to -1479 bp was identified. From a screen of wound-activated transcriptomic data, one transcriptional activator, IbbHLH3, and one repressor, IbbHLH4, were selected that bind to and activate or repress, respectively, the G-box motif in the *IbNAC1* promoter to modulate the IbNAC1-mediated response. In the early wound response, the IbbHLH3-IbbHLH3 protein complex binds to the G-box motif to activate *IbNAC1* expression. Thus, an elegant defense network is activated against wounding stress. Until the late stages of wounding, IbbHLH4 interacts with IbbHLH3, and the IbbHLH3-IbbHLH4 heterodimer competes with the IbbHLH3-IbbHLH3 complex to bind the G-box and suppress *IbNAC1* expression and timely terminates the defense network. Moreover, the JAZs and IbEIL1 proteins interact with IbbHLH3 to repress the transactivation function of IbbHLH3 in non-wounded condition, but their transcription is immediately inhibited upon early wounding. Our work provides a genetic model that accurately switches the regulatory mechanism of *IbNAC1* expression to adjust wounding physiology and represents a delicate defense regulatory network in plants.

## Author Summary

Plants develop sophisticated defense systems against insect attack. IbNAC1 is a transcriptional activator, which up-regulates *sporamin* expression against herbivory in sweet potato. However, it simultaneously injures the plant by IbNAC1-induced JA response. Thus, dynamic regulatory networks to coordinate the expression of *IbNAC1* are essential for appropriate growth and defense in sweet potato. Here, two bHLH transcription factors, IbbHLH3 and IbbHLH4, function antagonistically in activating the *IbNAC1* promoter. Notably, IbbHLH3 coordinates activity of *IbNAC1* promoter by interaction with several proteins in different time-course of wounding to activate/repress its transcriptional activation function. The dynamic interaction networks of IbbHLH3 improve defense ability against wounding, and timely prevent the injury by IbNAC1-regulated responses.

## Introduction

Wounding from insect herbivory is a common environmental stress that impacts agricultural productivity worldwide. Plants have developed sophisticated defense systems to cope with this frequent challenge. Among these systems, transcription factors play essential roles by modulating the expression of defense genes directly. Typically, transcription factors bind to a specific DNA motif in the promoter region of downstream genes based on their DNA binding domain. Several studies indicate that transcription factors have been characterized to bind with unusual *cis*-acting elements in promoter regions [1,2]. Following binding, several transcription factors regulate the promoter activity directly by activating or repressing the domain to control gene expression. To date, wound-activated promoters including *ATAF1/2* [3], *PI-II* [4], *WIPK* [5] and *sporamin* [6,7] have been identified. Thus, the dissection of the binding mechanism between transcription factors and the promoter region of defense genes is important for determining the defense response in plants.

Jasmonic acid (JA) is involved in plant defense against insect herbivory [8]. In response to wounding signals from herbivory, the elicited plant immediately synthesizes JA. After conjugation with Isoleucine (Ile), JA-Ile bound to the COI1 receptor induces the degradation of Jasmonate ZIM-domain (JAZ) proteins [9–12]. JAZ proteins repress MYC2/3/4 transcription factors, which play the critical role in regulating the defense response against herbivory, by forming the JAZ/MYC complex [13]. Upon JAZ degradation, MYC2-type transcriptional activators will activate their downstream targets by binding the G-box motif in the promoter regions of those genes [14,15]. Thus far, MYC2 has been identified to function as a positive regulator in the regulation of the direct defense genes *VSP1* and *VSP2* against herbivory. Interestingly, other MYC-type transcription factors, including JA-associated MYC2-like gene 1 (JAM1), JAM2 and JAM3, show repressive functions in herbivore defense, in contrast to MYC2 [16–18]. MYC2/3/4 and JAM1/2/3 belong to the basic helix-loop-helix (bHLH) transcription factor groups IIIe and IIId, respectively. These two subgroups of bHLH-III play antagonistic roles in several JA responses, including JA-induced leaf senescence, herbivore defense, anthocyanin accumulation, and other developmental processes, by competing for the binding site of the target promoter [16,17,19,20]. Additionally, both bHLH IIIe and IIId transcription factors readily form a homodimeric/heterodimeric complex with other proteins and function redundantly to regulate several JA responses, including anthocyanin accumulation, leaf senescence, and herbivory defense in plants [17, 20,21,22]. Based on these complicated regulatory mechanisms around bHLH transcription factors, plants can balance the response between herbivore defense and JA-induced injury.

Sweet potato is an important food crop, economic crop, and forage crop in Asia. Notably, it shows high resistance to insect feeding among crops in the field. A unique protein in sweet potato, sporamin, is abundant in tuberous roots as a storage protein for tuber sprouting [23]. In previous studies, sporamin has been found to function as a wound-inducible defense protein with strong activity against trypsin in leaves [24]. The overexpression of *sporamin* in *Brassica* and tobacco effectively increases resistance to insect feeding [25–27]. Thus, sporamin functions not only as a storage protein in tuberous roots but also as a wound-inducible defense protein in leaves. A wound-inducible NAC-domain transcription factor, IbNAC1, has been found to activate the *sporamin* promoter by binding the specific *cis*-acting element, TACAATATC, immediately upon wounding in leaves. In addition to enhanced resistance against herbivory, IbNAC1 simultaneously modulates several JA responses, including growth inhibition, ROS overproduction, and precocious senescence, to influence plant physiology [7].

In this study, we further investigate the signal transduction mechanism of wounding in the stimulation of *IbNAC1* expression. In the *IbNAC1* promoter, a G-box motif acts as a switch in response to wounding signals. Two bHLH MYC-type transcription factors, IbbHLH3 and IbbHLH4, were identified as binding proteins of the G-box switch in the *IbNAC1* promoter. These transcription factors function at different stages of the wounding response to synergistically coordinate *IbNAC1* expression. Furthermore, with the temporal changes that occur in response to wounding, the function of IbbHLH3 factor was activated or repressed through interactions with different proteins, including bHLHs, JAZs, EIL1, and MAPKs. This activation or repression of IbbHLH3 helps plants to cope with IbNAC1-mediated injury.

## Results

### Cloning and characterization of the *IbNAC1* promoter

**Fig 1:**
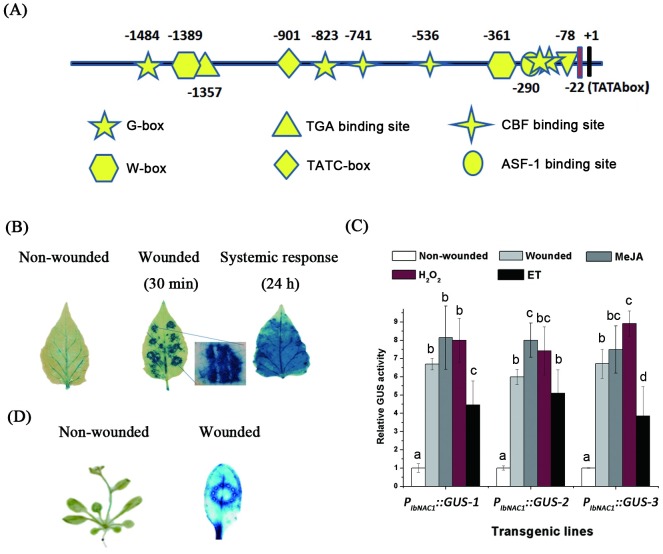
Cloning and characterization of the *IbNAC1* promoter. (A) A schematic feature of the *IbNAC1* promoter. Prediction of the *cis*-acting elements in the 1665-bp promoter region of *IbNAC1* was performed using the PLACE and PlantCare databases. (B) Activity analysis of the *IbNAC1* promoter upon wounding in transgenic sweet potato leaves. The GUS reporter gene driven by the *IbNAC1* promoter was transferred into sweet potato. Transgenic sweet potato leaves that had been mechanically wounded for 30 min using tweezers were stained with GUS staining buffer. The leaves were stained after 24 hours to analyze the systemic response of the *IbNAC1* promoter. (C) GUS activity in transgenic sweet potato with the GUS reporter gene driven by the *IbNAC1* promoter after wounding for 30 min or treatment with 50 μM MeJA for 1 hour, 10 mM ethephon for 1 hour, or 1% H_2_O_2_ for 1 hour. Error bars indicate standard deviations (SDs) (n = 10). Different letters represent significance as determined by one-way ANOVA (P<0.05). (D) GUS staining assay in transgenic Arabidopsis with the GUS reporter gene driven by the *IbNAC1* promoter. The wounded leaves were stained with GUS staining buffer after 30 min of treatment with a wheel.

Sweet potato IbNAC1 is known to reprogram the defense mechanism in response to herbivory and wounding [7]. However, the mechanisms of the wound signal response in gene expression remain ambiguous. To investigate the promoter function and control mechanism, a 1665-bp segment of the *IbNAC1* promoter region (upstream of the predicted transcriptional start site of *IbNAC1*) was isolated by genome walking (Clontech). Promoter structure analysis revealed that several *cis*-regulatory elements, including TATA-box, G-box motif, W-box motif, ASF-1 binding site, CBF binding site and LTRE-1 motif, were predicted by PLACE [28] and PlantCare [29] (Fig 1A). Furthermore, the promoter region was fused to the GUS reporter gene (*P*_*IbNAC1*_::*GUS*) and transformed into sweet potato and Arabidopsis. As shown in Fig 1B, strong signals of GUS activity were observed in local damaged leaf and remote undamaged leaves, suggesting that the *IbNAC1* promoter region was activated by both local and systemic wound responses. In addition to wounding-induced activity, several wounding-related signals, including methyl jasmonate (MeJA), ethylene (ET) and reactive oxygen species (ROS), also triggered the *IbNAC1* promoter (Fig 1C). These results correspond to the previously reported expression pattern of *IbNAC1* [7]. Simultaneously, transgenic Arabidopsis harboring *P*_*IbNAC1*_::*GUS* exhibited similar results with sweet potato under wounding treatment (Fig 1D), suggesting a similar signal transduction response to wounding to activate *IbNAC1* expression in sweet potato and Arabidopsis. In summary, the data suggest that the *IbNAC1* promoter region (1665 bp) harbors *cis*-acting element(s) for the wound response.

### Localization of wound-responsive *cis*-acting elements of the *IbNAC1* promoter region

**Fig 2:**
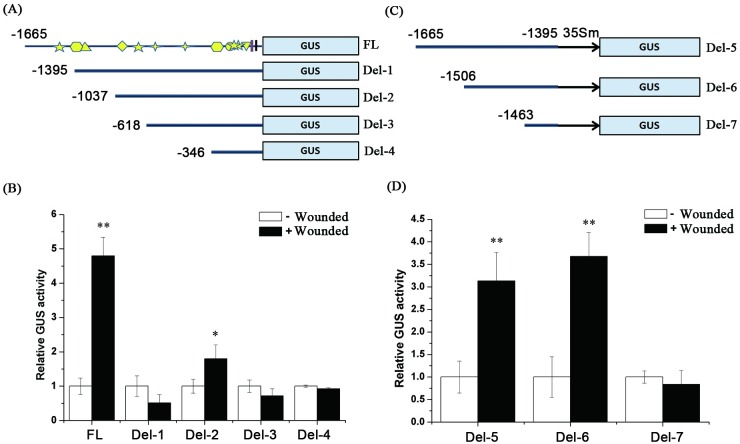
Position -1506 to -1463 bp (NWRE) determines the activity of the *IbNAC1* promoter in response to wounding. (A) A schematic model of the 5’ deletion of the primary promoter of *IbNAC1*. (B) GUS activity assay of each promoter fragment in transgenic Arabidopsis. Four independent lines of each construct were used to determine the GUS activity after wounding treatment for 30 min. Error bars indicate SDs from three independent replicates. Asterisks represent the significant differences from non-wounded plants by Student t-test’s (*, P<0.05; **, P<0.01). (C) A schematic model of a 5’ promoter deletion from -1665 to -1395 bp. (D) GUS activity assay of each promoter fragment fused to the 35S minimal promoter (35Sm) in transgenic Arabidopsis. Four independent lines of each construct were used to determine the GUS activity after wounding treatment for 30 min. Error bars indicate SDs from four independent replicates. Asterisks represent the significant differences from non-wounded plants by Student’s t-test (**, P<0.01).

To define the wound-responsive *cis*-regulatory element(s) in the *IbNAC1* promoter region, a 5’ serial deletion of the *IbNAC1* promoter was performed. Several truncated fragments of the *IbNAC1* promoter (FL, Del-1, Del-2, Del-3 and Del-4) fused to the GUS reporter were delivered into Arabidopsis plants to rapidly screen wound-responsive *cis*-elements (Fig 2A). Four independent T_4_ transgenic Arabidopsis lines were established for each deleted promoter construct to determine the promoter activity. FL (-1665 to -1 bp) transgenic plants exhibited obviously higher GUS activity in response to wounding. However, GUS activity was abolished in response to wounding in Del-1 (-1395 to -1 bp), Del-3 (-618 to -1 bp) and Del-4 (-346 to -1 bp) plants but not in Del-2 (-1037 to -1 bp) plants (Fig 2B). In Del-1 plants, the GUS activity dramatically decreased in response to wounding compared to that in FL plants. This indicates that the putative wound-responsive *cis*-element(s) were localized to the region from -1665 to -1395 bp. Furthermore, the promoter region (from -1665 to -1395 bp) with strong activity in response to wounding was dissected into three segments, -1665 to -1395 bp (Del-5), -1506 to -1395 bp (Del-6) and -1463 to -1395 bp (Del-7), and then fused to the 35S minimal promoter (35Sm) (Fig 2C). T_4_ transgenic lines with different constructs were selected to analyze GUS activity upon wounding. The results revealed that strong GUS activity in response to wounding was displayed in transgenic plants harboring Del-5 and Del-6 constructs but not the Del-7 construct (Fig 2D). This indicates that the DNA region from -1506 to -1463 bp is a wound-responsive *cis*-element in the *IbNAC1* promoter named NWRE (*Ib**N**AC1* Wound-responsive *cis*-Element).

### G-box has a wound-responsive function in the NWRE region of the *IbNAC1* promoter

**Fig 3:**
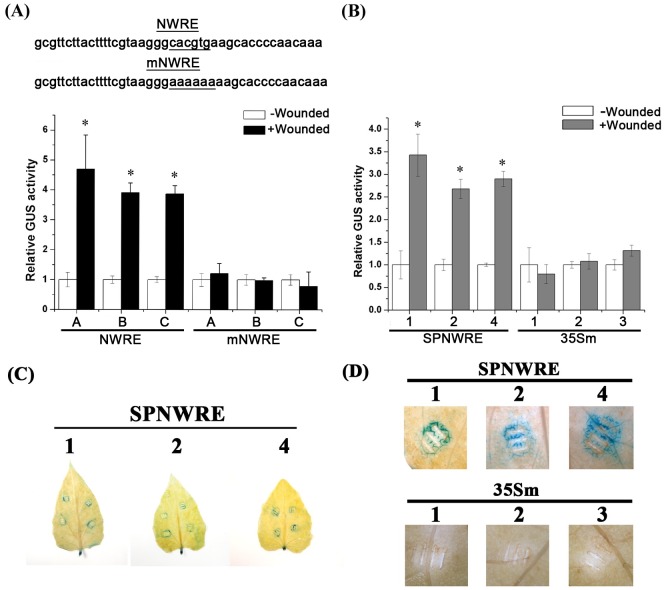
G-box within the NWRE plays an essential role in the wound response. (A) G-box plays an essential role in NWRE activation. The intact NWRE region or G-box-mutated NWRE region fused to the 35S minimal promoter (35Sm) and GUS reporter were transformed into Arabidopsis. The GUS activity was determined in three independent T_4_ transgenic lines under wound stress. Error bars indicate standard deviations (SDs) from four independent replicates. Asterisks represent significant differences from non-wounded plants by Student’s *t*-test (P<0.05). (B) NWRE region fused to 35Sm and the GUS reporter was transformed into sweet potato. Three independent transgenic lines were used to analyze the GUS activity under wounding. Transgenic lines containing *35Sm*::*GUS* were used as negative controls for wound activation. Error bars indicate SDs from four independent replicates. Asterisks represent significant differences from non-wounded plants by Student’s *t*-test (P<0.05). (C) Activity assay of NWRE in sweet potato leaves upon wounding. Three independent transgenic lines with *NWRE-35Sm*::*GUS* (SPNWRE) were wounded by tweezers. After wounding for 30 min, the wounded leaves were stained with GUS staining buffer at 25°C. (D) Microscopic observation of the wounded region in SPNWRE leaves after GUS staining. The wounded regions were monitored under a stereo microscope. *35Sm*::*GUS* transgenic lines (35Sm) were used as negative controls for GUS analysis.

To verify that the NWRE region (-1506 to -1463 bp) in the *IbNAC1* promoter is a wound-responsive region, the 43-bp fragment was amplified, fused to the 35Sm and the GUS reporter gene (*NWRE-35Sm*::*GUS*) and transformed into Arabidopsis. T_4_ NWRE transgenic plants were used to study the wound-responsive activity. As shown in Fig 3A, transgenic plants harboring the NWRE construct showed strong GUS activity in response to wounding. Based on the bioinformatics analysis, a wound-related *cis*-element (CACGTG), G-box, was found in the NWRE region. The G-box has been identified as a key motif in activating promoters in responses to wounding, JA and ABA [30]. To confirm whether the G-box in the NWRE region is responsible for wounding signal, a nucleotide mutational assay was performed. The G-box motif in the NWRE (CACGTG) was completely replaced with AAAAAA. The mutated NWRE (mNWRE) was fused to 35Sm and the GUS reporter (*mNWRE-35Sm*::*GUS*) and then transformed into Arabidopsis. Three different independent lines were selected to determine the GUS activity. In contrast to intact NWRE transgenic lines, the wounding-responsive activity was completely aborted in all of the mNWRE lines, suggesting that the G-box motif is essential for the wound signal response in the NWRE of the *IbNAC1* promoter (Fig 3A).

To further confirm the wound-responsive activity in sweet potato system, *NWRE-35Sm*::*GUS* was transformed into sweet potato. Three independent transgenic sweet potato lines with *NWRE-35Sm*::*GUS* showed strong GUS activity in the vicinity of the region wounded by tweezers compared to *35Sm*::*GUS* lines (Fig 3B to 3D). These results demonstrate that NWRE indeed plays a wound-responsive role in activating the *IbNAC1* promoter in sweet potato leaves.

### IbbHLH3 and IbbHLH4 bind to the G-box motif in the NWRE region of the *IbNAC1* promoter

**Fig 4:**
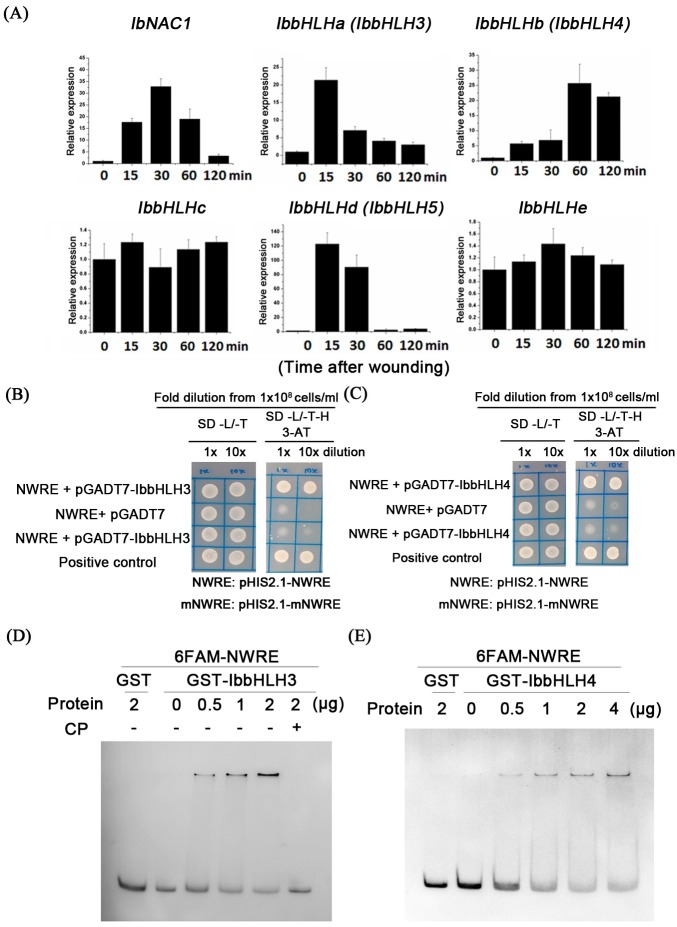
IbbHLH3 and IbbHLH4 physically bind to the NWRE region. (A) Expression levels of bHLH transcription factors in sweet potato leaves upon wounding. The bHLH transcription factors that were selected from the transcriptome dataset of wounded leaves were analyzed by a wounding time-course treatment. *IbNAC1* was shown as reference. Error bars indicate standard deviations (SDs) from four independent replicates (B) NWRE binding analysis. Yeast one-hybrid (Y1H) screening was used to analyze the binding ability of IbbHLHs with NWRE DNA *cis*-elements. NWRE fragments were cloned into the pHIS2.1 vector as bait. Then, the bait and pGADT7-IbbHLHs (AD-IbbHLHs) were co-transformed into yeast strain Y187. The growth of the yeast transformants is shown on both +His medium and -His medium with 100 mM 3-AT. The G-box-mutated NWRE was used as a control to verify the binding abilities of IbbHLH3 and IbbHLH4. (C) NWRE binding analysis of IbbHLH4 by Y1H screening. (D) NWRE binding analysis of IbbHLH3 by EMSA. GST-IbbHLH3 binds to 6FAM-NWRE in the presence of changing protein concentrations. The 50x unlabeled NWRE was included as a competitor (CP), and the GST protein was used as a negative control for NWRE binding. (E) NWRE binding analysis of IbbHLH4 by EMSA. GST-IbbHLH4 binds to 6FAM-NWRE depending on the IbbHLH4 protein concentration. The GST protein was used as a negative control for NWRE binding.

Previous studies have reported that the G-box motif can be bound physically by bHLH-type transcription factors [13] and bZIP-type transcription factor groups G and H [31]. Therefore, our wounding transcriptomic datasets [32] were carefully surveyed, and five bHLH transcription factors, namely *IbbHLHa*, *b*, *c*, *d* and *e*, were screened out. Subsequently, the gene expression pattern was monitored by qRT-PCR under a wounding time-course analysis. The data showed that the expression of only *IbbHLHa*, *IbbHLHb* and *IbbHLHd* was induced in wound-elicited leaves (Fig 4A). Among these genes, *IbbHLHa* and *IbbHLHb* (renamed IbbHLH3 and IbbHLH4, respectively) expression were correlated to *IbNAC1* expression pattern. Therefore, both were cloned for further investigation. IbbHLH3 and IbbHLH4 belong to groups IIIe and IIId of MYC2-related bHLH transcription factors, respectively (S1 Fig). A sequence analysis of IbbHLH3 and IbbHLH4 showed that the bHLH domain was conserved in IbbHLH3 and IbbHLH4, but the acidic region of MYC2/3/4, which acts as a transcriptional activation domain [33], was divergent in IbbHLH4 (S2 Fig). Interestingly, *IbbHLH3* and *IbbHLH4* showed different timely expression patterns under wounding stress. *IbbHLH3* was expressed in the early wound response. It peaked immediately within 15 min and then gradually decreased for 30 min. This expression pattern is corresponding to that of *IbNAC1* (Fig 4A). In contrast to *IbbHLH3* and *IbNAC1*, *IbbHLH4* was expressed during the late wound response. It was induced slightly during early wounding and then peaked at 60 min (Fig 4A). The two wound-inducible patterns of IbbHLH3 and IbbHLH4 suggest that these proteins may play different roles in controlling *IbNAC1* expression level upon wounding.

JA, salicylic acid (SA), ET and ROS are important elicitors that amplify the wound signal in plants. In our previous studies, *sporamin* and *IbNAC1* were reported to be triggered by JA, ET and H_2_O_2_ [6,7]. Thus, we monitored the expression of *IbbHLH3* and *IbbHLH4* under different elicitor treatments of wound-related events. Interestingly, these two bHLH transcription factors showed different expression patterns under different treatments. *IbbHLH3* expression was mainly triggered by JA and slightly induced by SA and H_2_O_2_. *IbbHLH4* expression was induced by JA, similar to that of *IbbHLH3*, but significantly increased in response to H_2_O_2_ and was not affected by SA treatment, suggesting that these two bHLHs may play different roles in controlling wound-induced ROS signaling (S3 Fig).

We then became interested in whether the NWRE region is bound directly by IbbHLHs. We tested for an interaction between the IbbHLHs and the NWRE by a yeast one-hybrid assay. In this assay (Fig 4B), the transformants co-expressing pGADT7-IbbHLH3 (*pADH1*::*AD-IbbHLH3*) and pHIS2.1-NWRE (*NWRE-TATAbox*::*HIS3*) were grown on SD-L/-T/-H medium with 100 mM 3-AT. This means that IbbHLH3 was capable of binding to the NWRE region of the *IbNAC1* promoter. To determine whether IbbHLH3 interacts with the G-box motif in the NWRE region, a G-box mutational assay was performed. In the mutated assay, the sequence of the G-box in the NWRE (GCGTTCTTACTTTTCGTAAGGGCACGTGAAGCACCCCAACAAA) was replaced with GCGTTCTTACTTTTCGTAAGGGAAAAAAAAGCACCCCAACAAA (mNWRE) and cloned into the pHIS2.1 vector (pHIS2.1-mNWRE). The transformant co-expressing pHIS2.1-mNWRE and pGADT7-IbbHLH3 was unable to grow on SD-L/-T/-H 3-AT medium. The absence of an interaction between mNWRE and IbbHLH3 indicated that IbbHLH3 binds to the NWRE region of the *IbNAC1* promoter via the G-box motif. Additionally, the transformants co-expressing pGADT7-IbbHLH4 (*pADH*::*AD-IbbHLH4*) and pHIS2.1-NWRE grew well, but a lack of growth of the transformants with pGADT7-IbbHLH4 and pHIS2.1-mNWRE on SD-L/-T/-H 3-AT medium was observed, suggesting that the *HIS3* gene driven by the NWRE was activated by the binding of AD-IbbHLH4 to the G-box motif (Fig 4C). For further confirmation of the binding activity between the NWRE DNA region and IbbHLH3/IbbHLH4, we conducted gel mobility assay by EMSA. The data agreed with those of the yeast one-hybrid assay (Fig 4D and 4E). The fluorescent NWRE probe was shifted by the addition of either IbbHLH3 or IbbHLH4, suggesting that both IbbHLH3 and IbbHLH4 can physically bind to the NWRE region.

### IbbHLH3 belongs to bHLH-IIIe group, and IbbHLH4 belongs to bHLH-IIId group

IbbHLH3 and IbbHLH4 belong to group III of bHLH-MYC type transcription factors (S1 Fig). The conserved nuclear localization signals (NLS) RPKKRGRKP and RPRKRGRKP, predicted by cNLS Mapper, were present in IbbHLH3 and IbbHLH4, respectively (S2 Fig). To test this prediction, the coding regions of IbbHLH3 and IbbHLH4 were cloned into the p2YGW7 vector, forming 3*5S*::*YFP-IbbHLH3* and *35S*::*YFP-IbbHLH4*, respectively. Both IbbHLH3 and IbbHLH4 were found under confocal microscopy to be nuclear proteins, similar to AtMYC2 (S4 Fig).

A phylogenetic tree analysis revealed that IbbHLH3 is closely related to group IIIe of bHLH transcription factor, which contains the transcriptional activators AtMYC2, AtMYC3 and AtMYC4 [14,34]. On the other hand, IbbHLH4 was classified into group IIId, which includes the transcriptional repressors JAMs (S1 Fig). To verify the roles of IbbHLH3 and IbbHLH4, we performed a transactivation reporter assay in yeast strain AH109 (*GAL*_*UAS*_*-TATA*::*LacZ*). IbbHLH3 and IbbHLH4 fused to the GAL4-binding domain (BD), forming BD-IbbHLH3 and BD-IbbHLH4, respectively, were transferred into AH109. After a β-GAL filter assay, visible blue precipitates were seen in BD-IbbHLH3, suggesting that IbbHLH3 functions as a transcriptional activator to activate *LacZ* reporter (S5A and S5B Fig). In contrast, the transformant expressing BD-IbbHLH4 could not activate LacZ reporter, similar to the transformant expressing BD alone, suggesting that IbbHLH3 and IbbHLH4 play different roles.

### IbbHLH3 and IbbHLH4 antagonistically regulate the *IbNAC1* promoter by competing to bind the NWRE

**Fig 5:**
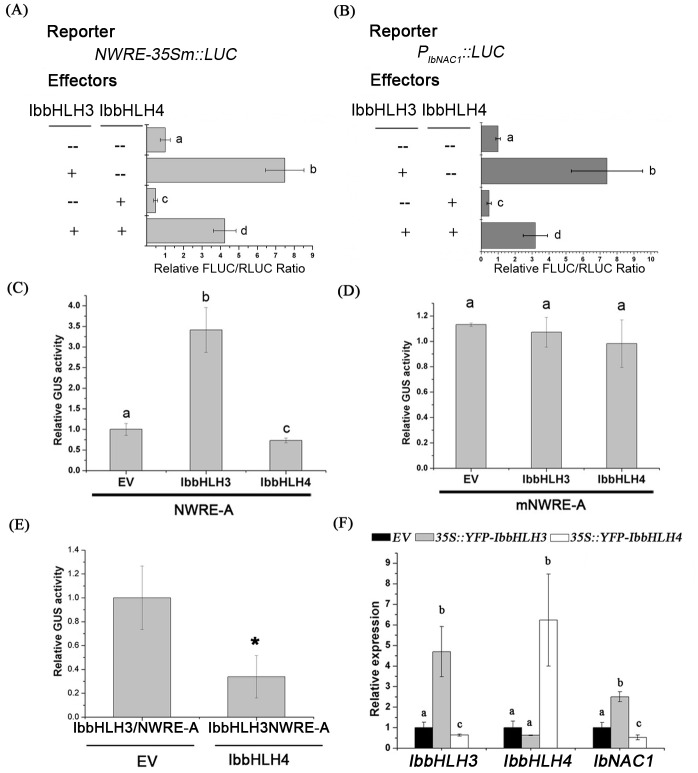
IbbHLH4 antagonizes IbbHLH3 to negatively regulate *IbNAC1*. (A) Transactivation assay indicates that the activation of the NWRE region in the IbNAC1 promoter by IbbHLH3 was suppressed by IbbHLH4. RLUC driven by the 35S promoter was used as an internal control. Error bars represent standard deviations (SDs) (n = 10). Different letters represent significance as determined by one-way ANOVA (P<0.05). (B) Transactivation assay indicates that the activation of the *IbNAC1* promoter by IbbHLH3 was suppressed by IbbHLH4. Error bars represent SDs (n = 10). Different letters represent significance as determined by one-way ANOVA (P<0.05). (C) *In vivo* transactivation assay. *35S*::*IbbHLH3* or *35S*::*IbbHLH4* was expressed in NWRE-A Arabidopsis. Total protein extracts from 21-day old plants were used for GUS activity analysis. Error bars represent SDs from four biological replicates. Different letters mean the significant difference determined by one-way ANOVA (P>0.05). (D) *In vivo* transactivation assay *35S*::*IbbHLH3* or *35S*::*IbbHLH4* was expressed in mNWRE-A Arabidopsis to examine the regulatory function of IbbHLH3 and IbbHLH4 on G-box in NWRE. Error bars represent SDs from three biological replicates. Different letters represent significance as determined by one-way ANOVA (P<0.05). (E) *In vivo* competing assay. *IbbHLH4* was transiently expressed in IbbHLH3/NWRE-A plants by agro-infiltration. Error bars represent SDs from four biological replicates. Asterisk represents the significant difference from IbbHLH3/NWRE-A plants (Student’s *t*-test, P<0.05). (F) Overexpression of *IbbHLH3* and *IbbHLH4* in sweet potato. *35S*::*YFP* (EV), *35S*::*YFP-IbbHLH3*, or *35S*::*YFP-IbbHLH4* was expressed in sweet potato leaves by particle bombardment. The expression level of *IbNAC1* was quantified by qRT-PCR. Error bars indicate SDs from three biological replicates. Different letters represent significance as determined by one-way ANOVA (P<0.05).

Based on the data presented here, IbbHLH3 and IbbHLH4 can bind to the G-box motif within the NWRE region. In order to determine the function of IbbHLHs in activating the NWRE region in plants, we performed a transactivation assay using the firefly LUC (FLUC gene) driven by NWRE-TATAbox (Fig 5A) or the full-size *IbNAC1* promoter (Fig 5B) as reporters and co-transformed each into Arabidopsis protoplasts with effector *35S*::*IbbHLH3* or *35S*::*IbbHLH4* (Fig 5A and 5B). As shown in Fig 5A, the IbbHLH3 effector increased LUC activity, while the IbbHLH4 effector repressed LUC activity, indicating that IbbHLH3 and IbbHLH4 play opposing roles in regulating the NWRE region. When IbbHLH3 and IbbHLH4 co-existed in the protoplasts, the LUC activity evidently decreased compared to that in the presence of IbbHLH3 alone. It seems that the function of IbbHLH3 is antagonized by that of IbbHLH4 (Fig 5A). The full-size *IbNAC1* promoter fused to FLUC was also used as a reporter for the transactivation assay. Similar to the NWRE region, the full-length promoter region was activated by IbbHLH3 but repressed by IbbHLH4, and both proteins were antagonistic (Fig 5B). To confirm whether G-box is a major DNA binding site in the *IbNAC1* promoter for IbbHLH activation, a base mutational assay was performed using the truncated *IbNAC1* promoter (Del-1; without the G-box motif) or mNWRE driving the *FLUC* gene as the reporter. The truncated *IbNAC1* promoter and mNWRE abolished most of the elicitation activity of IbbHLH3 and the repression activity of IbbHLH4 (S6A and S6B Fig). These results clearly indicate that both IbbHLH3 and IbbHLH4 bind to the G-box in the NWRE to synergistically coordinate the promoter activity of *IbNAC1*.

To validate the function of IbbHLH3/4 *in vivo*, *IbbHLH3* and *IbbHLH4* driven by 35S promoter were overexpressed in NWRE-A Arabidopsis plant, respectively. The GUS activity assay of double transgenic plants resulted that IbbHLH3 is a transcriptional activator of NWRE promoter in planta, while IbbHLH4 acts as a negative role (Fig 5C). In contrast, the GUS activities were not influenced in mNWRE-A Arabidopsis plants despite overexpressing *IbbHLH3* or *IbbHLH4* (Fig 5D). Combinations of the EMSA and transactivation assay clearly indicate that IbbHLH3 and IbbHLH4 bind to the G-box in the NWRE to synergistically coordinate the promoter activity. Furthermore, the *in vivo* binding competition of NWRE by IbbHLH3 and IbbHLH4 was examined. *IbbHLH4* was expressed in IbbHLH3/NWRE-A plants by agro-infiltration. As *in vitro* assay, IbbHLH4 suppressed the activity of NWRE, which was activated by IbbHLH3 (Fig 5E), suggesting that IbbHLH4 competes the function of IbbHLH3 to bind to NWRE in plants.

The functions of IbbHLH3 and IbbHLH4 in regulating *IbNAC1* expression were further studied in sweet potato. *35S*::*YFP-IbbHLH3* or *35S*::*YFP-IbbHLH4* was transiently expressed in sweet potato leaves by particle bombardment. The expression of *IbNAC1* was increased by presence of *IbbHLH3* (Fig 5F). In contrast, the expression of *IbNAC1* in *IbbHLH4*-overexpressing plants was repressed compared with that in the EV plants. This demonstrated that these wound-inducible bHLHs indeed act the antagonistic roles in controlling *IbNAC1* expression in sweet potato.

### Interaction between IbbHLH3 and IbbHLH4 inhibits the transactivation function of IbbHLH3

**Fig 6:**
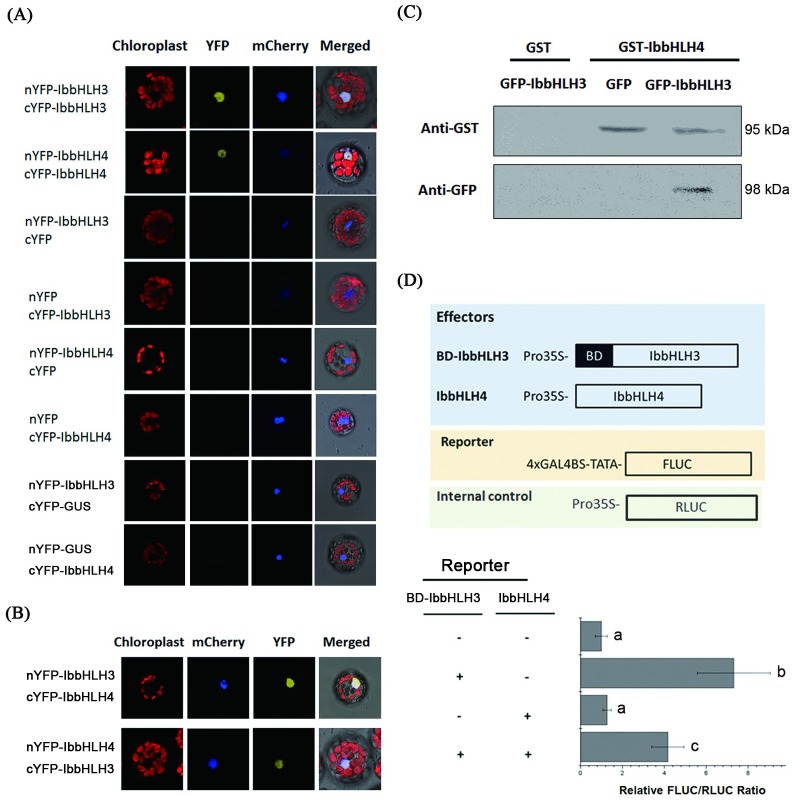
IbbHLH4 interacts with IbbHLH3 to repress the transcriptional activation function. (A) Bimolecular fluorescence complementation (BiFC) assay to detect the interactions of IbbHLH3 and IbbHLH4. The NLS-mCherry construct was co-expressed into protoplasts as a nuclei marker. The empty vectors of nYFP and cYFP interacted with IbbHLH3 and IbbHLH4, respectively, as negative controls. GUS protein was used as negative interaction protein of BiFC. (B) BiFC assay to detect the protein interaction between IbbHLH3 and IbbHLH4. (C) Co-IP assay to verify the interaction of IbbHLH3 with IbbHLH4 in plant. The 35S::GST-IbbHLH4 (GST-IbbHLH4) was co-expressed with 35S::GFP-IbbHLH3 (GFP-IbbHLH3) or 35S::GFP (GFP, control) in tobacco leaves. The total protein from the tobacco leaves were immunoprecipitated with the GST resin, and were further analyzed by western blot using anti-GST antibody and anti-GFP antibody. 35S::GST (GST) was used as negative control for IbbHLH3 interaction. (D) Transactivation assay using the GAL4DB-based expression system. The activation of Gal4BS by BD-IbbHLH3 binding was suppressed by IbbHLH4, while IbbHLH4 could not affect the activity of Gal4BS. RLUC driven by the 35S promoter was used as internal control to normalize the transfection efficiency. Error bars mean standard deviations (SDs) (n = 10). Different letters mean statistically significance determined by one-way ANOVA (P<0.05).

MYC-type bHLH transcription factors function in the formation of protein dimers depending on their HLH/ZIP domain in the C-terminal region to activate gene transcription [35]. Based on the sequence analysis, both IbbHLH3 and IbbHLH4 contain the conserved HLH domain and show high similarity to each other (S2 Fig), suggesting that they can form homodimeric/heterodimeric complexes. As shown in Fig 6A, the bimolecular fluorescence complementation (BiFC) assay demonstrated that both IbbHLH3 and IbbHLH4 interact with themselves to form a protein complex (Fig 6A). Additionally, the BiFC (Fig 6B), Co-IP (Fig 6C) and MBP pull-down assay (S7 Fig) results demonstrated that IbbHLH3 can interact with IbbHLH4 as a heterodimer. This interaction between bHLH-IIId and bHLH-IIIe suggests a unique strategy in regulating the downstream genes in sweet potato.

The results of transactivation assay in Fig 5 demonstrate the positive regulatory role of IbbHLH3 and the negative role of IbbHLH4 in regulating the NWRE region and the full-length *IbNAC1* promoter. We are interested in how the heterodimeric complex of IbbHLH3-IbbHLH4 regulates *IbNAC1* promoter activity. Therefore, we generated a chimeric protein BD-IbbHLH3 by fusing the GAL4 DNA binding domain (BD) with IbbHLH3, which can bind to Gal4BS DNA sequence. The Gal4DB transactivation expression assay was carried out by interacting two effectors, *35S*::*BD-IbbHLH3* and *35S*::*IbbHLH4*, and one reporter, *4xGAL4BS-TATA*::*FLUC*. In this transient activation system, the enhanced reporter activity by IbbHLH3 was decreased by the co-expression with IbbHLH4 (Fig 6D). However, IbbHLH4 did not affect the reporter activity directly. These results reveal that IbbHLH4 suppresses the reporter activity by inhibiting the transactivation function of IbbHLH3. To validate whether these interactions of IbbHLH3 coupled with its transactivation function, a truncated IbbHLH3 protein lacking the HLH domain, which is a critical region for interaction with bHLHs in group IIIe [35], was created. The result revealed that the truncated IbbHLH3 was unable to activate the transcription of *IbNAC1* in sweet potato (S8 Fig). It suggested that the interaction ability of IbbHLH3 with itself or other IbbHLH proteins determines its function in regulating *IbNAC1* expression.

Furthermore, we generated a truncated IbbHLH4 protein (ΔIbbHLH4; S9A Fig), which is modified by deleting 10 amino acids from the basic DNA binding motif [13]. The truncated IbbHLH4 loses DNA-binding activity to the NWRE region (S9B Fig) but retains its interaction with the IbbHLH3 protein, as demonstrated by BiFC (S9C Fig). The transactivation analysis between ΔIbbHLH4 and IbbHLH3 revealed that LUC activity was suppressed as well, compared to that in IbbHLH3-IbbHLH3 (S9D Fig). These data clearly indicate that IbbHLH4 and IbbHLH3 can form a heterodimeric complex that effectively abolishes the promoter regulatory activity of IbbHLH3.

### IbbHLH3 interacts with JAZs, EIL1, and MAPKs during wounding

**Fig 7:**
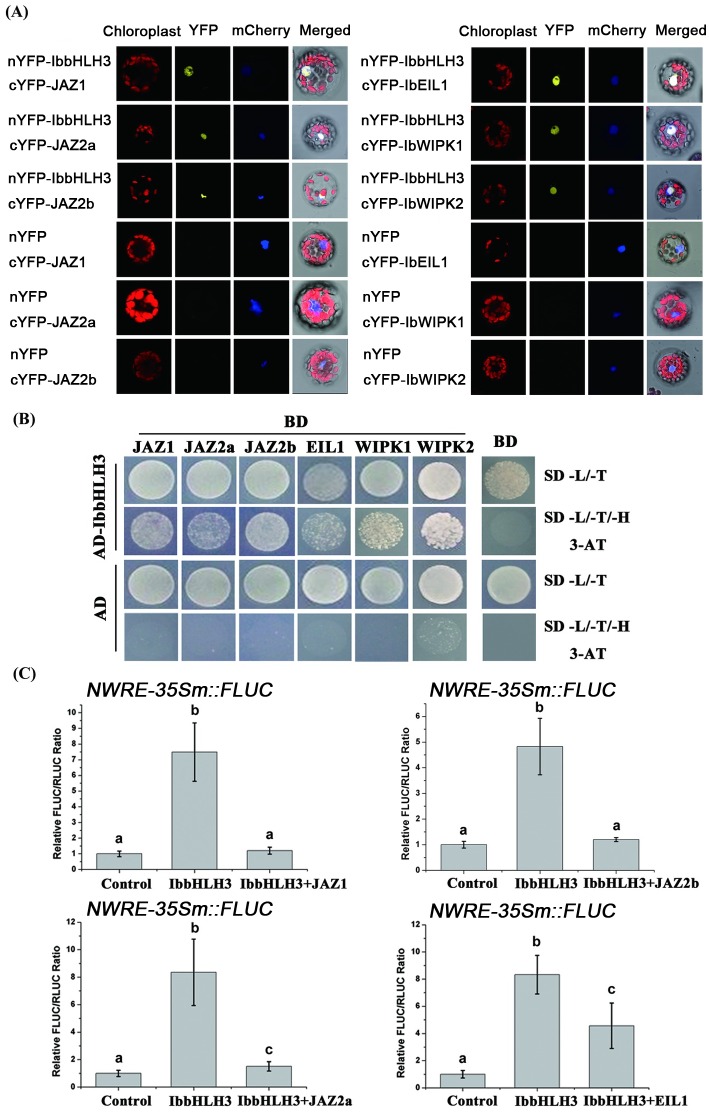
The protein interaction of IbbHLH3 with, JAZs, IbEIL1 and MAPKs. (A) BiFC assay to detect the heterodimeric interaction of IbbHLH3 with JAZs (IbJAZ1, IbJAZ2a, and IbJAZ2b), IbEIL1, and IbMAPK (IbWIPK1 and IbWIPK2). The NLS-mCherry construct was co-expressed into protoplasts as a nuclear marker. (B) Yeast two-hybrid assays. The interactions were indicated by the ability of yeast transformants to grow on SD medium lacking Leu/Trp/His (SD -L-T/-H) with 5 mM 3-AT. The coding region of IbbHLH3 was cloned into pGADT7 (AD), and IbJAZs, IbEIL1, and IbWIPKs were cloned into pGBKT7 (BD). Empty vector was used as negative control for IbbHLH3 interaction. (C) Competing assay of IbbHLH3. The activation of NWRE by the expression of *IbbHLH3* was inhibited by the co-expression with *JAZ1*, *JAZ2a*, *JAZ2b* and *IbEIL1*. Error bars mean standard deviations (SDs) (n = 10). Different letters represent the significance determined by one-way ANOVA (P<0.05).

bHLH-MYC-type proteins interact with several proteins, including bHLH, JAZ and MYB, altering the transcriptional regulatory activities. In this study, the ability of MYC-type IbbHLH3 to bind to target proteins was confirmed. As is known, the JA ZIM-domain protein (JAZ) family proteins repress JA signaling by interacting with MYC2/3/4 transcription factors. Here, we are interested in determining the function of wound-responsive JAZs genes in sweet potato leaves. Using transcriptomic analysis, several wound-related JAZ proteins were selected to examine the interaction with IbbHLH3. Among these genes, the expression of *JAZ2a* and *JAZ2b* were rapidly repressed in the early wound response. In contrast, the expression of *JAZ1* was induced late by wounding (S10 Fig). To examine the potential interaction between wound-responsive JAZs and IbbHLH in sweet potato, a BiFC assay was performed in Arabidopsis protoplasts. As shown in Fig 7A, an YFP signal in the nucleus was seen with the co-expression of nYFP-IbbHLH3 with cYFP-JAZ1, cYFP-JAZ2a, or cYFP-JAZ2b. Further, we conducted yeast two-hybrid assays (Y2H) to verify these interactions (Fig 7B). The growth of yeast transformants on SD medium lacking Leu/Trp/His indicated that IbbHLH3 can interact with JAZs. Protein pull-down assay was also performed to retest the interaction abilities. The maltose binding protein (MBP)-fused IbbHLH3 (MBP-IbbHLH3) could be pulled down by GST-JAZs (S11 Fig). These results evidently suggested that IbbHLH3 interacts with JAZ proteins. Based on interaction results between the IbbHLH3 and JAZs, we assumed that may influence the transactivation activity of IbbHLH3. As expected, the competing assay showed that the regulatory activity of IbbHLH3 in the enhanced NWRE region was suppressed by co-expression with JAZs, suggesting that JAZ proteins interact with IbbHLH3 to attenuate IbbHLH3 activity (Fig 7C).

In addition to JAZ proteins, several wound-related IbbHLH3 target proteins were also screened in this study. A wound-repressible EIN-like gene (S10 Fig), *IbEIL1*, was cloned and used for BiFC and Y2H. The BiFC results revealed that a strong YFP signal was reconstructed by nYFP-IbbHLH3 and cYFP-IbEIL1 in the cell nucleus (Fig 7A). The interaction between IbbHLH3 and IbEIL1 was simultaneously evidenced by Y2H (Fig 7B). As shown above, the formation of the IbbHLH3 complex would potentially repress the function of IbbHLH3 to activate the NWRE promoter region. Thus, we were interested in determining whether the transactivation function of IbbHLH3 was influenced by interaction with IbEIL1. The protoplast transactivation assay showed that IbbHLH3 significantly activated LUC activity driven by the NWRE. However, IbEIL1 evidently suppressed LUC activity by forming IbEIL1-IbbHLH3 (Fig 7C). Additionally, two wound-inducible MAPK family genes, IbWIPK1 and IbWIPK2, in sweet potato were also identified here to interact with IbbHLH3 (Fig 7A and 7B and S11 Fig). The sequence analysis of IbbHLH3 revealed that the phosphorylated sequence [33,36] was present in IbbHLH3 (S12 Fig). Thus, it is assumed that these wound-related MAPKs may regulate protein activity or the stabilization of IbbHLH3 by phosphorylation during wounding stress.

## Discussion

Sporamin, a unique herbivore defense protein in sweet potato, plays a critical biochemical role in response to insect feeding. Its expression is regulated by the IbNAC1 transcription factor in sweet potato leaves under wound stress. Previously, we reported that the IbNAC1 protein is a wound-inducible transcriptional activator by binding to the SWRE *cis*-element of the *sporamin* promoter to initiate *sporamin* expression [7]. Our present work indicates that the transcription activity of *IbNAC1* is antagonistically regulated by the activator IbbHLH3 and the repressor IbbHLH4 to activate the defense network in sweet potato leaves upon wounding.

### G-box motif affects the activity of the *IbNAC1* promoter in response to wounding

Several NAC-domain proteins have been found to participate in the JA-mediated defense response in Arabidopsis, including ANAC019 and ANAC055 in the NAC3 subfamily of NAC-domain transcription factor [37]. Zheng et al. [38] reported that the MYC2 transcription factor can bind to the promoter regions of *ANAC019* and *ANAC055* to regulate transcription. However, the transcription factors regulating the NAC-domain ATAF-group proteins remain unknown. In this study, we started to isolate the wound-responsive *cis*-acting element in the promoter region of *IbNAC1*, which belongs to the ATAF subfamily of NAC-domain proteins. Although several G-box motifs were present in the *IbNAC1* promoter (Fig 1A), only the G-box localized within the NWRE from -1484 to -1479 bp in the promoter region responded to stress signals, including wounding, H_2_O_2_ and JA (Figs 2 and 3 and S3 Fig). This result demonstrates that the G-box (from -1484 to -1479 bp) in the *IbNAC1* promoter region acts as a core switch in response to wound signaling. The G-box motif has been reported to affect the activity of several promoters in response to multiple stimuli, such as light, ABA, MeJA and wounding [39,40] Williams et al. [41] reported that the flanking sequences of G-box affect the specificity of protein binding. Indeed, the sequences flanking at G-box from -1484 to -1479 bp differed from those flanking sequences of other G-box motifs in the *IbNAC1* promoter. The G-box sequence, CACACGTGGG, localized within the Del-4 region of the *IbNAC1* promoter (Fig 2A), has been reported that does not interact with the G-box-binding protein [41]. Thus, the flanking sequences of G-box may directly affect the activity in response to wounding. Although the G-box motif is a major role in response to wounding, the GUS activities in Del-2 plants were slightly activated by wounding (Fig 2A). This implied that the other factors may co-regulate the *IbNAC1* promoter in addition to the IbbHLH3/4. W-box has been identified as either wound-activated or wound-repressed DNA motif in plant [42]. Thus, we speculated that the W-box within Del-1 region may act as a repressor to interfere the activation ability of Del-2 in response to wounding.

### Antagonistic function of IbbHLH3 and IbbHLH4

Several bHLH transcription factors function as either transcriptional activators or transcriptional repressors for the regulation of downstream genes. IbbHLH3 and IbbHLH4 belong to bHLH groups IIIe and IIId, respectively (S1 Fig). Essentially, group IIIe contains MYC-type transcriptional activators, e.g., MYC2/3/4; whereas, group IIId MYC-type factors function as transcriptional repressors, e.g., JAM1/2/3. To analyze the activation domain contained in IbbHLH3 and IbbHLH4, a transactivation assay was conducted. The GAL4DB-based expression system is a perfect method for determining the transcription activation ability of transcription factors [43]. IbbHLH3 acted as transcriptional activator, similar to MYC2; whereas, IbbHLH4 could not activate the reporter (S5 Fig). Consistent with the yeast activation assay, IbbHLH3 and IbbHLH4 exhibited antagonistic functions in the regulation of the *IbNAC1* promoter (Fig 5). Generally, transcriptional repressors can be classified as active or passive. Active repressors directly influence the transcription of downstream gene by harboring a repression domain, such as the EAR motif [44]. Although IbbHLH4 can repress the activity of the NWRE promoter region, it does not possess a known transcriptional repression domain (Fig 5). Therefore, we conclude that IbbHLH4 is likely to be a passive repressor by interfering with the activator. Consistent with its repressive mode, IbbHLH4 bound to the NWRE region by IbbHLH4-IbbHLH4 protein complex (Figs 5A and 5B and 6A) or interfered with the IbbHLH3 transactivation function (Fig 6D) by forming a IbbHLH3-IbbHLH4 heterodimer (Fig 6B and 6C). Additionally, IbbHLH3-ΔIbbHLH4 and IbbHLH3 protein lacking the HLH domain showed the insufficient affinity to bind to and activate NWRE (S8 and S9 Figs), suggesting that the NWRE activity was only activated by IbbHLH3-IbbHLH3. Collectively, IbbHLH3 and IbbHLH4 act in opposition in response to wound signaling and antagonistically control the promoter activity of *IbNAC1*.

Notably, bHLH-IIIe group activators can interact with each member in group IIIe to form homodimers or heterodimers in Arabidopsis [14]. However, the direct interaction of bHLH-IIIe members (transcriptional activators) with bHLH-IIId members (transcriptional repressors) has never been reported in Arabidopsis [17]. Interestingly, the current work clearly shows that IbbHLH4 of bHLH-IIId acts as a transcriptional repressor to antagonize the transcriptional activity of IbbHLH3 of bHLH-IIIe both by promoter binding competition and by direct protein-protein interaction (Figs 5 and 6). This result indicated that further investigation was needed to determine the differences between Arabidopsis and sweet potato in coordinating the interactions of bHLH. The HLH domain in bHLH transcription factors is required to form homo- or heterodimers with other bHLH transcription factors [14,35]. However, the HLH domain in IbbHLH3 and IbbHLH4 is conserved with groups IIIe and IIId in Arabidopsis (S12 and S13 Figs). However, the flanking sequences of HLH-domain in IbbHLH3 were distinct from Arabidopsis bHLH-IIIe proteins. This divergence may affect the interaction ability with bHLH-IIId proteins in plants.

### Protein-protein interaction of IbbHLH3 influences its transactivation function

The core proteins in JA signaling, JAZs, interact with the bHLH-IIIe and -IIId transcription factors, including MYC2/3/4 and JAM1/2/3, forming a complex to repress their activation function in non-wounded plants [14,17,18,45]. Here, we identified some wound-responsive JAZ proteins as targets of IbbHLH3. These JAZ proteins can inhibit the transactivation function of IbbHLH3 in the NWRE DNA region by forming a protein complex (Fig 7). When sweet potato leaf is exposed to wound stress, the expression of *JAZ2a* and *JAZ2b* was repressed in the early stages of wounding (S10 Fig). This activates IbbHLH3 to regulate *IbNAC1* expression, activating the genetic defense network against herbivory attack. In the late response to wounding, the elevated *JAZ1* transcripts were able to suppress the function of IbbHLH3, similar to IbbHLH4.

A previous study showed that the interaction between AtMYC2 and AtEIN3 antagonistically modulates JA and ethylene signaling. AtEIN3 interacts with and inhibits the transcriptional activation function of AtMYC2. The repression function of AtMYC2 inhibits the expression of herbivore-defensive genes that are enhanced by JA [22]. In this study, an EIN3-like gene, IbEIL1, was found to interact with IbbHLH3. Consistent with the model in Arabidopsis, the protein complex of IbbHLH3-IbEIL1 also lost the transactivation ability of IbbHLH3 (Fig 7c). Notably, *IbEIL1* is a wound-repressible gene in sweet potato leaves. In the early stages of the wound response, *IbEIL1* was repressed immediately until 2 hours post wounding (S10 Fig), indicating that wounded sweet potato would restrain the expression of *IbEIL1* and *JAZ2* in the early wound response to allow IbbHLH3 to function. In addition, we found that wound-related MAPK3/6 interacted with IbbHLH3 in this study (Fig 7a). Zhai et al. [33] demonstrated that the phosphorylation of MYC2 is important for JA signaling in plant immunity. Through an LC-MS/MS analysis, they identified a phosphorylated site at Thr328 (P(pT)P). Furthermore, SDHS, a phosphorylation site in MYC2 [36], was also identified in IbbHLH3 (S12 Fig). Recently, MYC2 has been reported to interact with and be phosphorylated by MPK6 in Arabidopsis under blue light treatment [46]. However, the mechanism of MYC2 phosphorylation in wounding remains largely unknown. These studies may explain our finding that MAPK proteins interact with IbbHLH3.

### Coordination of antagonistic and synergistic mechanism for the wound response in sweet potato plants

**Fig 8:**
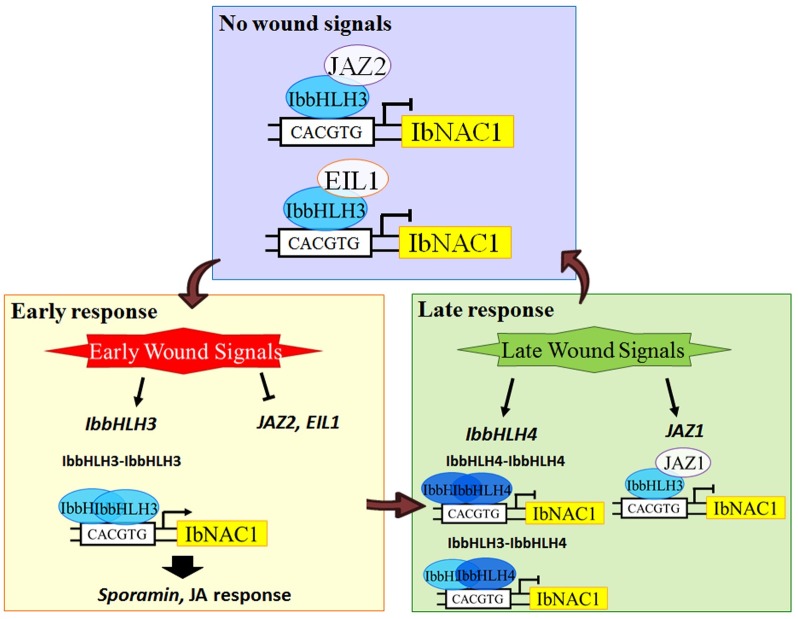
A proposed model in sweet potato leaves of wound signaling regulating *IbNAC1* expression against herbivory. The early wounding signals trigger the expression of *IbbHLH3* and repress the expression of *JAZs* and *EIL1*, which are repressors of IbbHLH3 that inhibit its transactivation function before wounding stress. The released IbbHLH3 activates the expression of *IbNAC1* by binding to the G-box motif in the promoter region. Subsequently, IbNAC1 up-regulates the expression of the *sporamin* gene and participates in the JA response against insect feeding. IbbHLH4 and JAZ1, induced in the late wound response, repress *IbNAC1* by competing with IbbHLH3 to avoid IbNAC1-mediated injury.

Collectively, this study reveals a model underlying the antagonism between the early wound response and the late wound response in sweet potato leaves (Fig 8). The antagonistic and synergistic mechanism of the wound response in plants helps to protect plants against herbivory and stabilizes the physiological changes that occur in response to elicitation. Several studies have indicated that the expression level of JA-related defense genes is highly up-regulated in response to insect attack. However, there were costs associated with this defense-responsive mechanism, including accelerated senescence, ROS overproduction, and growth and development inhibition [7,16,19,47,48]. Thus, the antagonistic role of timely switching off the wound-responsive genetic network against herbivory attack is important for plant. In our previous study, we identified IbNAC1, which regulates the expression of *sporamin* and is involved in the JA response [7]. Although IbNAC1 effectively controls the herbivory defense mechanism by regulating *sporamin* expression, it was suppressed after early wounding to avoid JA-mediated injury due to IbNAC1 hyper-activation. Our present study proposed an integrated mechanism of wound signal transduction. The coordinated regulation of the wound response by the transcriptional activator IbbHLH3 and the transcription repressor IbbHLH4 represents an appropriate strategy for defense during wound stress.

## Materials and Methods

### Plant materials

Sweet potato (*Ipomoea batatas* cv. Tainong 57) was grown at 25°C/20°C with Light/Dark photoperiod of 16 h/8 h. The third leaves of sweet potato were wounded using tweezers. For hormone treatment, the third expended leaves were sprayed with 0.1 M sodium phosphate buffer or sodium phosphate buffer containing treated chemicals (0.05 mM MeJA, 1% H_2_O_2_, 10 mM ethephon and 2 mM SA) at 25°C for 1 hour.

### Promoter construction

To construct a reporter vector to analyze the regulatory *cis*-element in the *IbNAC1* promoter in response to wounding, the promoter region (1665 bp; FL) and the consecutively truncated regions, such as Del-1 (1395 bp), Del-2 (1037 bp), Del-3 (618 bp) and Del-4 (346 bp), amplified by PCR were respectively cloned into pCAMBIA1391z to drive the GUS reporter gene. Furthermore, the selected region from -1665 to -1395 bp was further divided into three different fragments named Del-5 (-1665 to -1395 bp), Del-6 (-1506 to -1395 bp), and Del-7 (-1463 to -1395 bp). These fragments were respectively fused to the 35Sm promoter and GUS reporter. In parallel, the 35Sm promoter fused to the GUS reporter was used as a negative control in the wounding treatment. These reporter constructs were transformed into Arabidopsis and sweet potato plants to analyze the promoter activity.

### RNA extraction and qRT-PCR analysis

The treated leaves of sweet potato were ground under liquid nitrogen by pestle. The extraction method was as described by Chang et al. [49]. A total of 3 μg of total RNA was performed for first-strand cDNA synthesis using M-MuLV reverse transcriptase (Fermentas, Waltham, MA, USA) with dT primer. Real-time qPCR analysis was conducted with cDNA using the SYBR FAST qPCR kit (KAPA, USA). *IbACTIN7* (S1 Table) was selected as an internal control to normalize the gene expression.

### Plant transformation

Sweet potato callus was induced from scion, and then the suspended callus cells were infected with *Agrobacterium tumefaciens* strain EHA105 [50]. The transformants were screened using 10 ppm hygromycin for 28 days. The floral dipping method [51] was used to transform the constructs into *Arabidopsis thaliana* Col-0.

### Analysis of GUS activity

The treated transformants with the GUS reporter were fixed with formaldehyde and immersed in GUS staining buffer as described by Plesch et al. [52]. For GUS chemical activity analysis, 4-MUG (Sigma, USA) as substrate was reacted with total solution protein from treated plants at 37°C to analyze GUS activity. The fluorescence in the reaction mix was determined using an Infinite M200 plate reader (filter: excitation 355 nm, emission 460 nm).

### Yeast one-hybrid assay

The 43-bp NWRE fragment amplified by PCR was inserted into pHIS2.1 (Clontech, CA, USA) to generate the plasmid pHIS2.1-NWRE as bait. The coding regions of IbbHLH3 and IbbHLH4 were separately cloned into pGADT7 as effectors. Lithium acetate-mediated transformation [53] was performed to transform the constructs into yeast strain Y187. In a preliminary nutrient deficiency screening, the transformants were grown on SD/-Leu/-Trp medium (Clontech) at 30°C for 4 days. The surviving transformants were further selected using SD/-Leu/-Trp/-His medium with 100 mM 3-AT (3-amino-1,2,4-triazol; Sigma, USA) at 30°C for 4 days.

### Transactivation assay in yeast system

IbbHLHs proteins were examined for transactivation function using a yeast system. The coding region of IbbHLH3 and IbbHLH4 were cloned into pBGKT7 (BD). Then, the BD-IbbHLH3 and BD-IbbHLH4 were delivered into yeast strain AH109. The transformants were screened by tryptophan-deficient SD medium, and determined its reporter activity by a filter assay using X-gal (Sigma, USA) as substrate.

### Purification of GST-recombinant proteins

The open reading flame of IbbHLH3 and IbbHLH4 was cloned behind the GST-tag in the pGEX4T-3 vector (GE Healthcare, Sweden). The fusion plasmids were transferred into *E*. *coli* Cordon. The GST fusion proteins in bacterial cultures were induced by 0.1 mM IPTG for 4 hours. The cell pellets were lysed, and the recombinant proteins were purified using GSTrap FF (GE Healthcare, Sweden). Then, the concentration of the extracted proteins was determined by the Bradford method [54].

### Electrophoretic mobility shift assays (EMSA)

Single strands of NWRE fused to 6FAM were synthesized (Sigma, USA). The two strands of NWRE were mixed together in equimolar ratios and reacted at 95°C and cooled slowly to 25°C. The double-stranded NWRE fused to 6FAM were used as fluorescent probes for the EMSA experiment.

The EMSA binding reaction contained appropriately 5X binding buffer (50 mM pH 8.0 Tris HCl, 750 mM KCl, 2.5 mM EDTA, and 62.5% glycerol), 1 μg of poly (dI-dC), a 6X dye, 1 μl of 6FAM-labeled NWRE probe, and added proteins in dosage-dependent manner. The reaction mixtures were reacted at room temperature, and then loaded into a 6.6% native bis-polyacrylamide gel in 0.5X TBE buffer. The shift of the probes in the gels was observed using LAS3000.

### Phylogenetic analysis

The amino acid sequences were retrieved from GenBank. Multiple sequence alignments of the bHLH-MYC domain proteins were conducted using the MEGA 4.1 program. Phylogenetic analyses were generated by the neighbor-joining method. A bootstrap analysis of 1000 resampling replications was performed in MEGA 4.1.

### Subcellular localization

The p2YGW7 vector, which contains the 35S promoter, was used to drive the YFP-IbbHLH fusion proteins. Fluorescence images were obtained by confocal microcopy (Leica TCS SP5).

### Protoplast transient activation assay

The NWRE region and the *IbNAC1* promoter fused to a TATA box and firefly LUC, respectively, were used as reporter plasmids. The reporter plasmids (5 μg) and the effector plasmids (5 μg), *35S*::*IbbHLH3* and *35S*::*IbbHLH4*, were used for protoplast transactivation analysis. In the competing assay, an extra 5 μg of competitors were co-transformed with effectors and reporters. 2 μg of reference plasmid (RLUC) was co-transformed for normalization of each reaction, after which the LUC activity was examined by a dual-luciferase assay system (Promega, USA).

### *In vivo* transactivation assay

The coding region of *IbbHLH3* and *IbbHLH4* was amplified by PCR, and subsequently cloned into pCAMIBA1300, which harbored a 35S promoter. These plasmid constructs were delivered into T_4_ NWRE-A plants and T_4_ mNWRE-A plants, respectively. T_3_ double transgenic plants were used to determine the NWRE activity by GUS reporter assay.

### Particle bombardment

*35S*::*YFP*, *35S*::*YFP-IbbHLH3* or *35S*::*YFP-IbbHLH4* were delivered by using GDS-80 gene delivery system (Wealtec, USA). Gold particles (0.4 μm diameter) coated with 1 μg plasmid DNA were shot by 50 psi of helium, the gas flow rate around 10~15 L/min, and 6 cm target distance. After bombardment for 3 days, the expression of *IbbHLH3*, *IbbHLH4*, and *IbNAC1* were monitored by qRT-PCR.

### BiFC assay

Full-length coding sequences of sweet potato IbbHLH3, IbbHLH4, JAZ1, JAZ2a, JAZ2b, IbEIL1, IbWIPK1, and IbWIPK2 were cloned into the nYFP or cYFP vector through a Gateway reaction with the pSAT4-DEST-nEYFP and pSAT4-DEST-cEYFP vector systems [55]. The constructs were transiently expressed in Arabidopsis protoplast cells, and the YFP signal was monitored by confocal microscopy.

### Co- immunoprecipitation assay

The plasmid constructs *pCAMBIA1300-35S*::*GST-IbbHLH4* and *pCAMBIA1300-35S*::*GFP-IbbHLH3* were co-transformed into *Agrobacterium tumefaciens* LBA4404. The acetosyringone-activated *Agrobacterium* with was injected into *N*. *benthamiana* leaves. Total proteins from the transformation leaves were incubated with GST-Resin (GenScript, USA). After washing, the bound proteins were eluted and detected by western blotting using anti-GFP (Genscript, USA), and anti-GST antibodies (Genscript, USA).

### Protein pull-down assay

The recombinant protein GST-IbbHLH4 or GST was co-incubated with MBP-IbbHLH3 and amylose resin (Biolab, South Africa). After washing five times with MBP column buffer, the MBP-binding protein was eluted and separated by SDS-PAGE. Anti-MBP and anti-GST antibodies were used for western blotting analysis to detect the MBP-IbbHLH3 and IbbHLH3-interacting proteins (GST-IbbHLH4 or GST). For GST pull-down assay, the GST-fusion proteins (GST-JAZs and GST-MAPKs) were co-incubated with MBP-IbbHLH3 and GST-Resin (GenScript, USA). After washing with PBS buffer, the GST-binding protein was eluted and detected by western blotting using anti-GST and anti-MBP antibodies (GenScript, USA).

### Yeast two-hybrid assay

The coding region of IbbHLH3 was inserted into pGADT7 to generate the plasmid AD-IbbHLH3. The coding regions of IbJAZs, IbWIPKs, and IbEIL1 were separately cloned into pGBKT7 (BD; Clontech, CA, USA). Lithium acetate-mediated transformation [50] was performed to transform the constructs into yeast strain AH109. The transformants were grown on SD/-Leu/-Trp medium at 30°C for 4 days, and the surviving transformants were further selected using SD/-Leu/-Trp/-His medium with 5 mM 3-AT at 30°C for 4 days.

### Statistical analysis

The experiments in this study were examined at least three individual biological replicates. Student’s *t*-test was used to determine the statistical significance of the experiments in Excel 2010 at P<0.05 and P<0.01. Statistical analysis of one-way ANOVA was performed to determine the significant differences in SPSS at P<0.05.

### Accession numbers

Gene sequences from present study can be retrieved at GenBank/EMBL as following: IbNAC1 (GQ280387), IbbHLH3 (KU589265), IbbHLH4 (KU744525), IbJAZ1 (KX147234), IbJAZ2a (KX147232), IbJAZ2b (KX147233), IbEIL1 (KX147235), IbWIPK1 (HQ434622), IbWIPK2 (KU744642), AtMYC2 (AT1G32640), AtMYC3 (AT5G46760), AtMYC4 (AT4G17880), AtJAM1 (AT1G01260), AtJAM2 (AT1G01260), and AtJAM3 (AT4G16430).

## Supporting Information

S1 Table Primer list. (PDF)

S1 Fig Phylogenetic relationship of IbbHLH3 and IbbHLH4 with related transcription factors. The phylogenetic relationships were analyzed by the neighbor-joining method. A bootstrap analysis of 1000 resampling replications was conducted in MEGA 4.1.
(TIF)

S2 Fig Sequence alignment analysis of IbbHLH3 and IbbHLH4 with MYC-domain bHLH proteins. The sequences of IbbHLH3, IbbHLH4 and bHLH-MYC-type transcription factors, including AtMYC2, AtMYC3, AtMYC4, and JAM1, were aligned by GeneDoc. The transcriptional activation domain is shown in the gray region, and the underlined region is the bHLH-conserved domain. NLS are marked by a red frame.
(TIF)

S3 Fig Expression levels of *IbbHLH3* and *IbbHLH4* under wound-related signaling. The expression levels of IbbHLH3 and IbbHLH4 were monitored by qRT-PCR under treatments of 50 μM MeJA for 1 hour, 2 mM SA treatment for 1 hour, and 1% H2O2 treatment for 1 hour. Error bars indicate SDs from three biological replicates.
(TIF)

S4 Fig Subcellular localization of IbbHLH3 and IbbHLH4. IbbHLH3 and IbbHLH4 were cloned into the p2YGW7 vector, forming *35S*::*YFP-IbbHLH3* and *35S*::*YFP-IbbHLH4*, respectively. Simultaneously, AtMYC2 was used as a positive control for nuclear fluorescence observation under confocal microscopy.
(TIF)

S5 Fig Transactivation of IbbHLH3 and IbbHLH4 by the yeast Gal4BD system. (A) IbbHLH3 and IbbHLH4 were cloned into the yeast expression vector pGBKT7, forming BD-IbbHLH3 and BD-IbbHLH4, respectively. The yeast AH109 transformants containing BD-IbbHLH3 and BD-IbbHLH4 were analyzed by a β-GAL filter assay. (B) Expression analysis of the LacZ gene in EV, BD-IbbHLH3, and BD-IbbHLH4 transformants. Error bars indicate SDs (n = 5).
(TIF)

S6 Fig Activation assay of IbbHLH3 and IbbHLH4 using the truncated promoter of *IbNAC1* and the mutated NWRE region. The truncated IbNAC1 promoter (Del-1) (A) and the mutated NWRE region with 35Sm (B) were separately fused to FLUC as reporters. Renilla LUC (RLUC) was used as an internal control for normalization. Error bars indicate SDs (n = 10).
(TIF)

S7 Fig MBP pull-down assays for the interaction between IbbHLH3 and IbbHLH4. GST–IbbHLH4 or GST was incubated with MBP-IbbHLH3 and amylose resin. The reacted proteins were eluted from the resin and examined by western blotting using anti-MBP or anti-GST antibody, respectively.
(TIF)

S8 Fig Interactions of IbbHLH3 with bHLH proteins determine its transactivation function. (A) A schematic representation of HLH domain in IbbHLH3. (B) The expression of *IbNAC1* and *IbbHLH3* in sweet potato overexpressing *IbbHLH3ΔHLH*. *35S*::*YFP-IbbHLH3ΔHLH* or *35S*::*YFP* was transiently expressed in sweet potato leaves by particle bombardment. After bombardment for 3 days, the expression of *IbNAC1* and *IbbHLH3* were monitored by qRT-PCR. Error bars indicate SDs (n = 5). Asterisks represent significant difference from YFP (Student’s *t*-test; P<0.05).
(TIF)

S9 Fig Competing assay of IbbHLH3 with the truncated DNA-binding domain of IbbHLH4. (A) A schematic model of the truncated DNA binding domain of IbbHLH4 (ΔIbbHLH4). (B) NWRE binding ability of truncated IbbHLH4. ΔIbbHLH4 was cloned into the yeast expression vector pGADT7, forming AD-ΔIbbHLH4. pHIS2-NWRE was used as bait to examine the binding ability of ΔIbbHLH4. (C) BiFC assay. ΔIbbHLH4 was fused to cYFP in the N-terminal region, and the interaction with IbbHLH3 analyzed in Arabidopsis protoplasts. (D) IbbHLH3 competing assay of ΔIbbHLH4 in regulation of the NWRE activity. Error bars indicate SDs (n = 10). Asterisks represent significant difference from reporter alone (*, P<0.05; **, P<0.01).
(TIF)

S10 Fig Expression pattern analysis of putative wound-related genes interacting with IbbHLH3 upon wounding. The expression of putative IbbHLH3-binding factors, including *JAZ1*, *JAZ2a*, *JAZ2b*, *EIL1*, *IbWIPK1*, and *IbWIPK2*, was monitored by qRT-PCR under a wounding time-course analysis. Error bars indicate SDs from four independent replicates.
(TIF)

S11 Fig GST pull-down assay for the interaction between IbbHLH3 and JAZs or MAPKs. The purified MBP-IbbHLH3 protein were incubated with the GST-fusion proteins (GST-JAZ1, GST-JAZ2a, GST-JAZ2b, GST-IbWIPK1 and GST-IbWIPK2), respectively. Bound proteins were precipitated with the anti-GST agarose and further analyzed by western blot using anti-GST antibody and anti-MBP antibody. The input lane represents the protein level of IbbHLH3 before reaction.
(TIF)

S12 Fig Sequence comparison of IbbHLH3 with AtMYC2/3/4. The amino sequence alignment of bHLH group IIIe transcription factors, including AtMYC2/3/4 and IbbHLH3, was analyzed by GeneDoc software. The HLH-domain (blue line) was conserved in bHLH-IIIe transcription factors. The putative phosphorylation site in IbbHLH3 was labeled as red underline.
(TIF)

S13 Fig Sequence comparison of IbbHLH4 with AtJAM1/2/3 and AtbHLH14. The amino sequence alignment of bHLH group IIId transcription factors, including AtJAM1/2/3 and IbbHLH4, was analyzed by GeneDoc software. The HLH-domain (blue line) was conserved in bHLH-IIId transcription factors, expect for AtbHLH14.
(TIF)
