# Supplementary material for: The Sweet Potato NAC-Domain Transcription Factor IbNAC1 Is Dynamically Coordinated by the Activator IbbHLH3 and the Repressor IbbHLH4 to Reprogram the Defense Mechanism against Wounding
Source: PLoS Genet. 2016 Oct 25;12(10):e1006397. doi: 10.1371/journal.pgen.1006397 (PMC5079590; doi:10.1371/journal.pgen.1006397)
Supplement: S1 Table — (PDF) [file pgen.1006397.s001.pdf]

**Primer name**	**sequence**
ACTIN7 FP	TTTGCTGGTGATGATGC
ACTIN7 RP	GCAACATACATGGCAGG
JAZ2a RT F	AATTCCTTCAGGTGTTTGCACAATT
JAZ2a RT R	CACACCTTTTCCATGAAGAAGTTGG
JAZ2b RT F	AATTCCTTCAGTTGCCGCTAAG
JAZ2b RT R	CACACCTTTTCCATGAAGAAGTTGG
JAZ1 RT F	CTGTTTCAAAGGTTGTCCCTTCTTTT
JAZ1 RT R	TCAGTGCTCAATTTTGACAGGAAAATG
IbEIL1 RT F	CGGGAGGTGGAATGTTTCAG
IbEIL1 RT R	TCAGAAGTACCATGTGGATCC
IbbHLH3 RT F	AACTCCTCCTCCTCTTCCGC
IbbHLH3 RT R	TTATAGTACCCATCGCCCCACC
IbbHLH4 RT F	ATTCGGAGATTCAAAACCACCAA
IbbHLH4 RT R	TTATAACCCAACAACAGATGACAGC
IbNAC1 RT F	CGGCCGGGGATACAAATTTGTAAGCTT
IbNAC1 RT R	GAATCGGAATCCCGGCGGCATCTC
IbWIPK1 RT F	CGAACGAGATGGTTGCGATTAA
IbWIPK1 RT R	GCTCCTCTGATAAACCTTGATTTGAT
IbWIPK2 RT F	GCCCGGAGATATGTTAGACAAC
IbWIPK2 RT R	TCAATAAGCTGGATCTGGGTTG
