# Supplementary figures and images for: The Sweet Potato NAC-Domain Transcription Factor IbNAC1 Is Dynamically Coordinated by the Activator IbbHLH3 and the Repressor IbbHLH4 to Reprogram the Defense Mechanism against Wounding
Source: PLoS Genet. 2016 Oct 25;12(10):e1006397. doi: 10.1371/journal.pgen.1006397 (PMC5079590; doi:10.1371/journal.pgen.1006397)

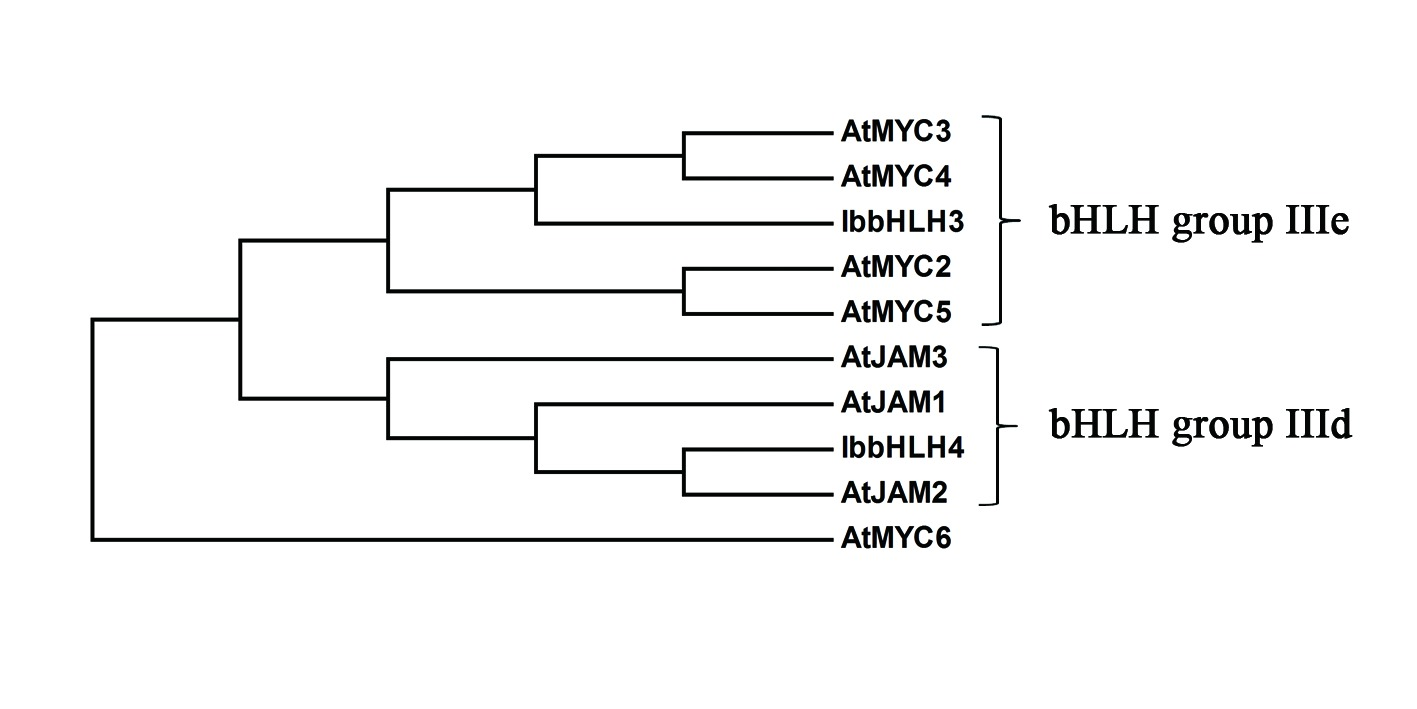

Supplement: S1 Fig — The phylogenetic relationships were analyzed by the neighbor-joining method. A bootstrap analysis of 1000 resampling replications was conducted in MEGA 4.1. (TIF) [file pgen.1006397.s002.tif]

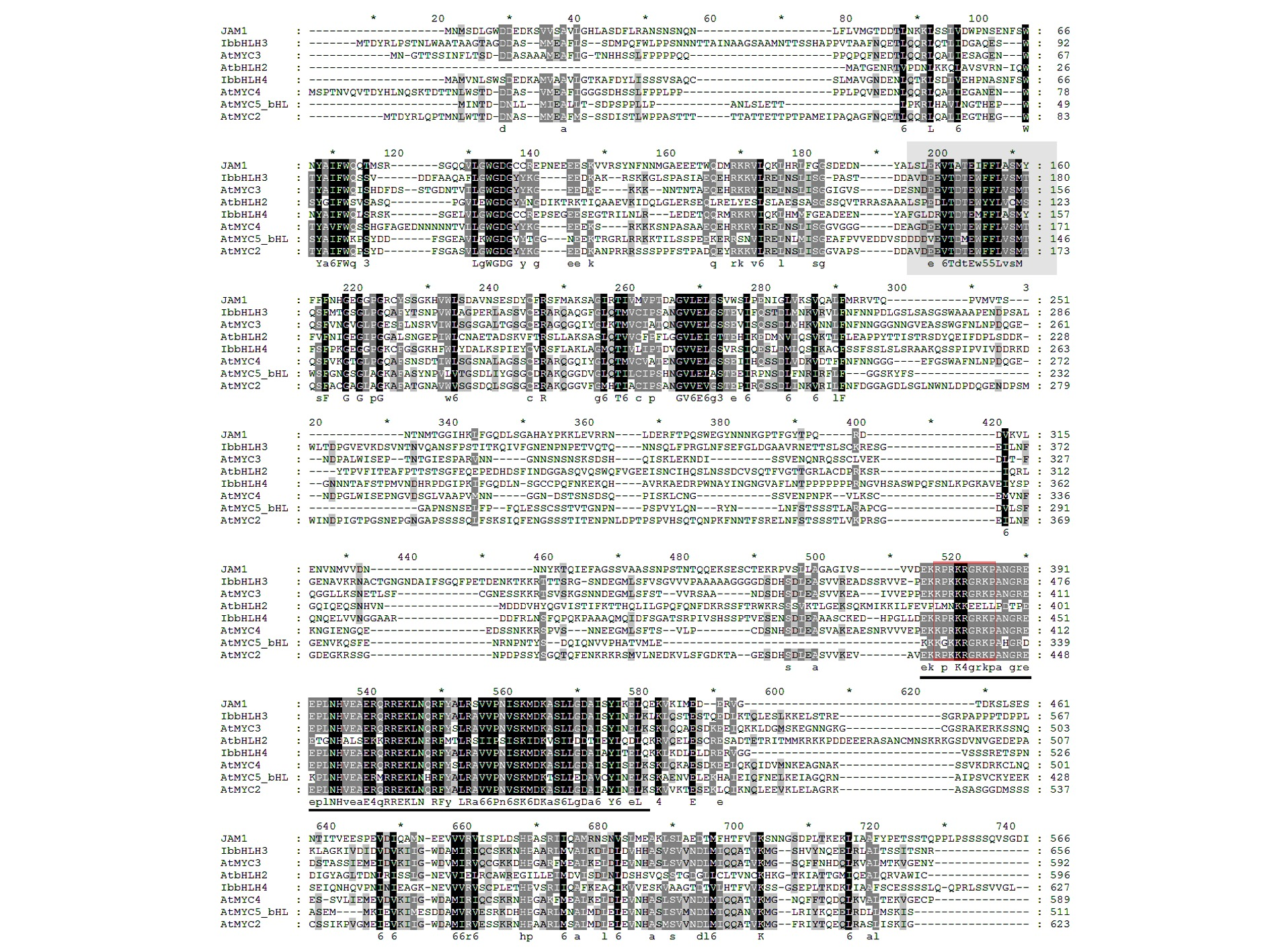

Supplement: S2 Fig — The sequences of IbbHLH3, IbbHLH4 and bHLH-MYC-type transcription factors, including AtMYC2, AtMYC3, AtMYC4, and JAM1, were aligned by GeneDoc. The transcriptional activation domain is shown in the gray region, and the underlined region is the bHLH-conserved domain. NLS are marked by a red frame. (TIF) [file pgen.1006397.s003.tif]

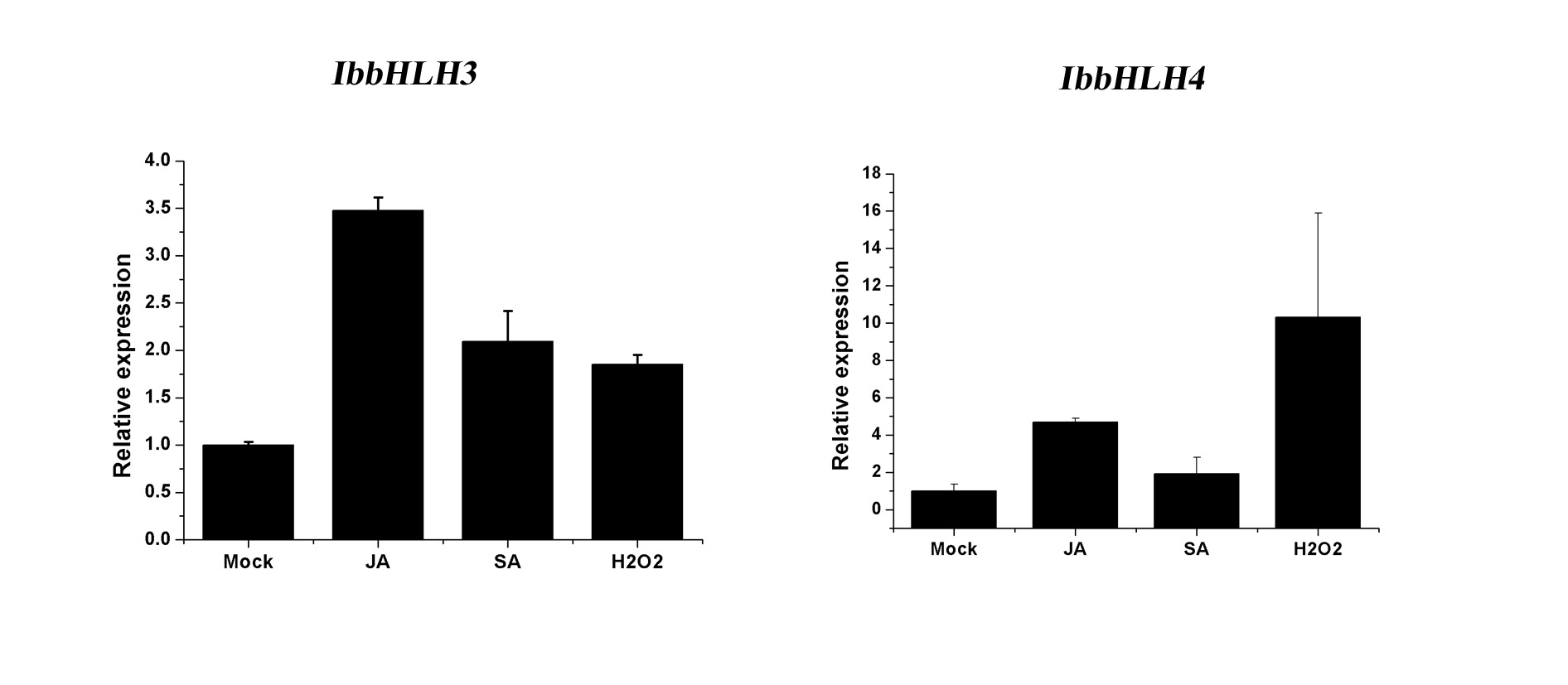

Supplement: S3 Fig — The expression levels of IbbHLH3 and IbbHLH4 were monitored by qRT-PCR under treatments of 50 μM MeJA for 1 hour, 2 mM SA treatment for 1 hour, and 1% H2O2 treatment for 1 hour. Error bars indicate SDs from three biological replicates. (TIF) [file pgen.1006397.s004.tif]

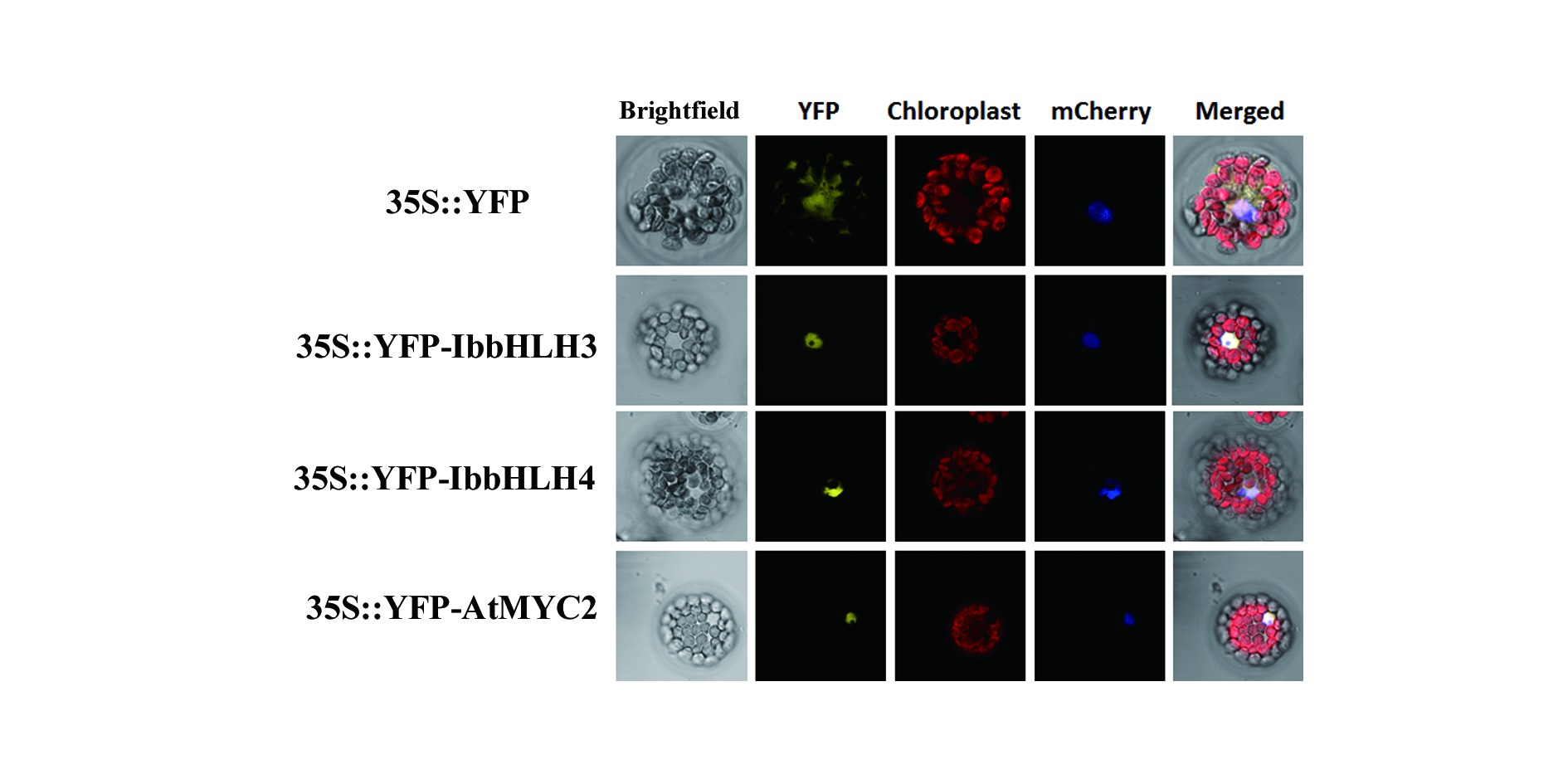

Supplement: S4 Fig — IbbHLH3 and IbbHLH4 were cloned into the p2YGW7 vector, forming 35S::YFP-IbbHLH3 and 35S::YFP-IbbHLH4, respectively. Simultaneously, AtMYC2 was used as a positive control for nuclear fluorescence observation under confocal microscopy. (TIF) [file pgen.1006397.s005.tif]

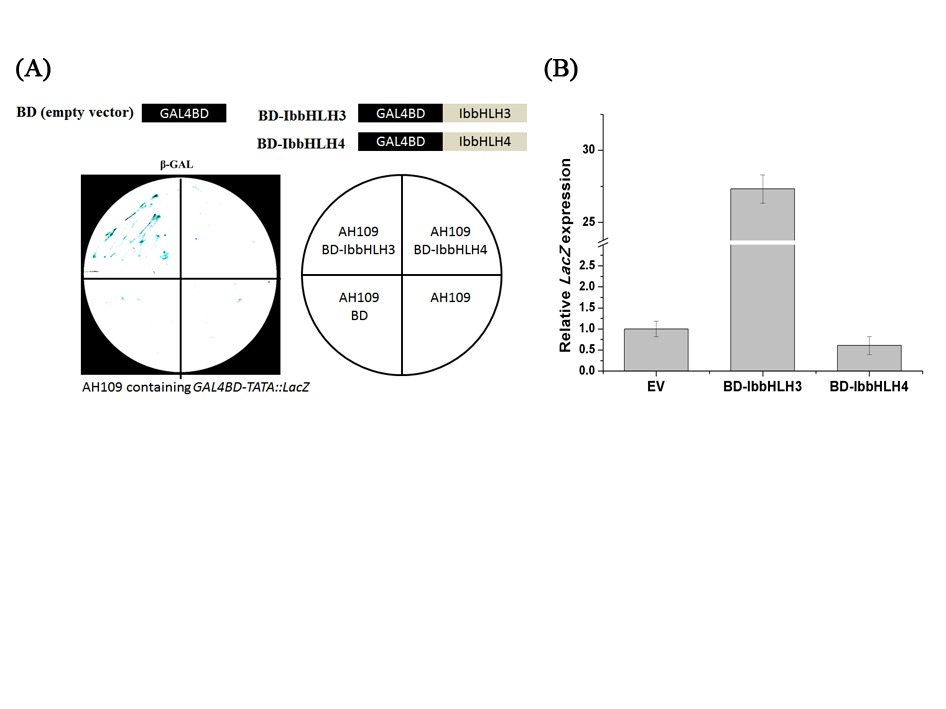

Supplement: S5 Fig — (A) IbbHLH3 and IbbHLH4 were cloned into the yeast expression vector pGBKT7, forming BD-IbbHLH3 and BD-IbbHLH4, respectively. The yeast AH109 transformants containing BD-IbbHLH3 and BD-IbbHLH4 were analyzed by a β-GAL filter assay. (B) Expression analysis of the LacZ gene in EV, BD-IbbHLH3, and BD-IbbHLH4 transformants. Error bars indicate SDs (n = 5). (TIF) [file pgen.1006397.s006.tif]

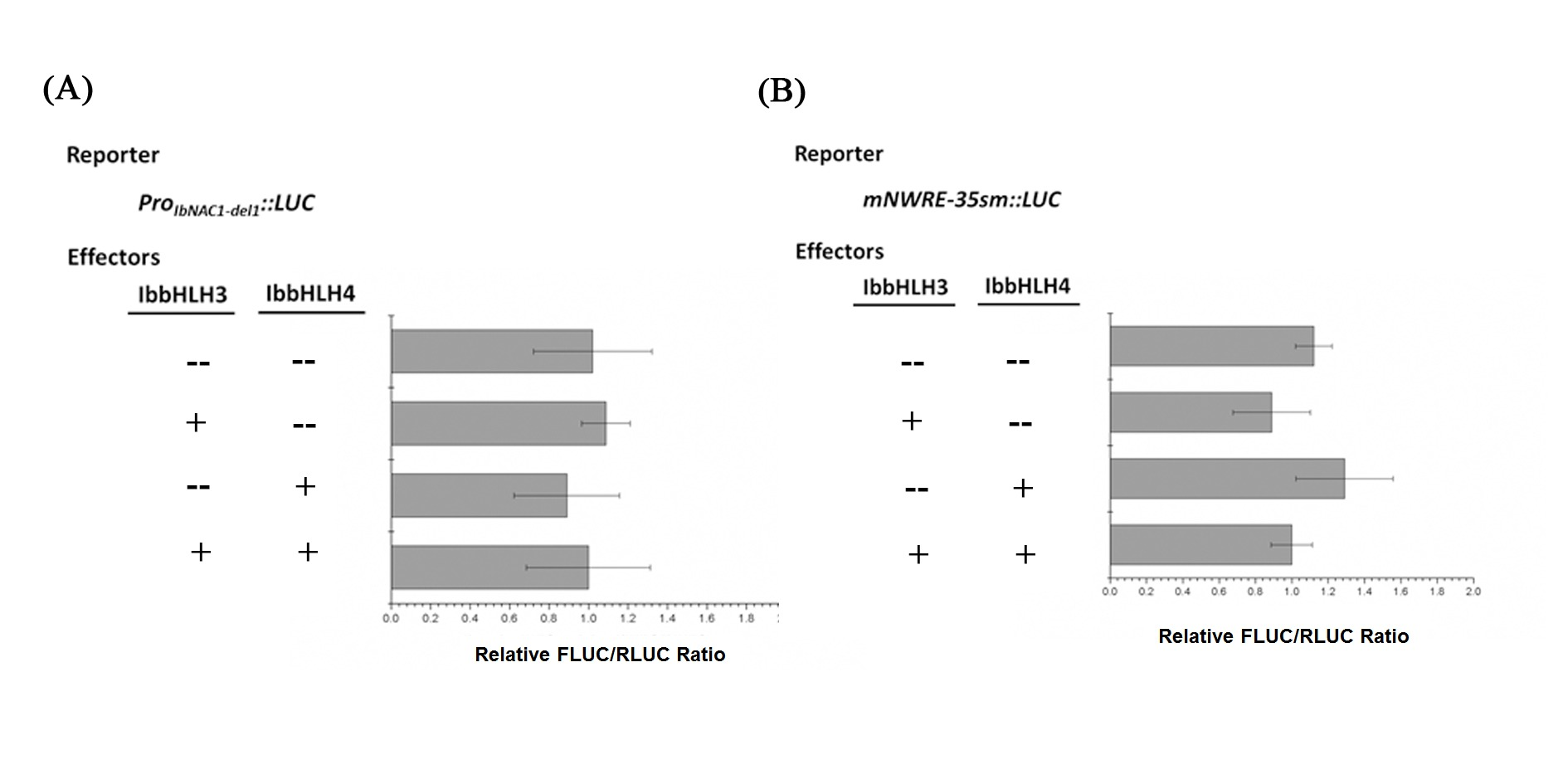

Supplement: S6 Fig — The truncated IbNAC1 promoter (Del-1) (A) and the mutated NWRE region with 35Sm (B) were separately fused to FLUC as reporters. Renilla LUC (RLUC) was used as an internal control for normalization. Error bars indicate SDs (n = 10). (TIF) [file pgen.1006397.s007.tif]

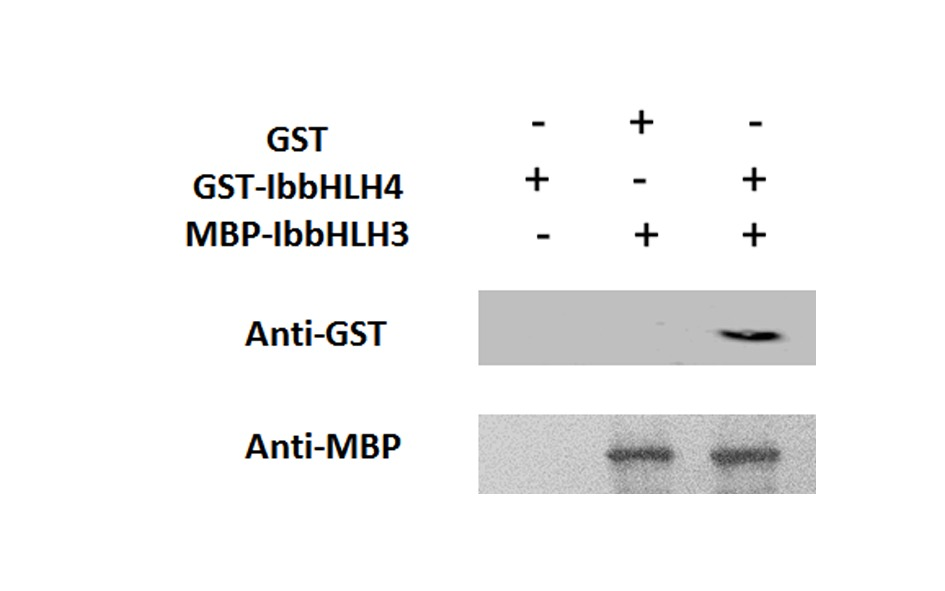

Supplement: S7 Fig — GST–IbbHLH4 or GST was incubated with MBP-IbbHLH3 and amylose resin. The reacted proteins were eluted from the resin and examined by western blotting using anti-MBP or anti-GST antibody, respectively. (TIF) [file pgen.1006397.s008.tif]

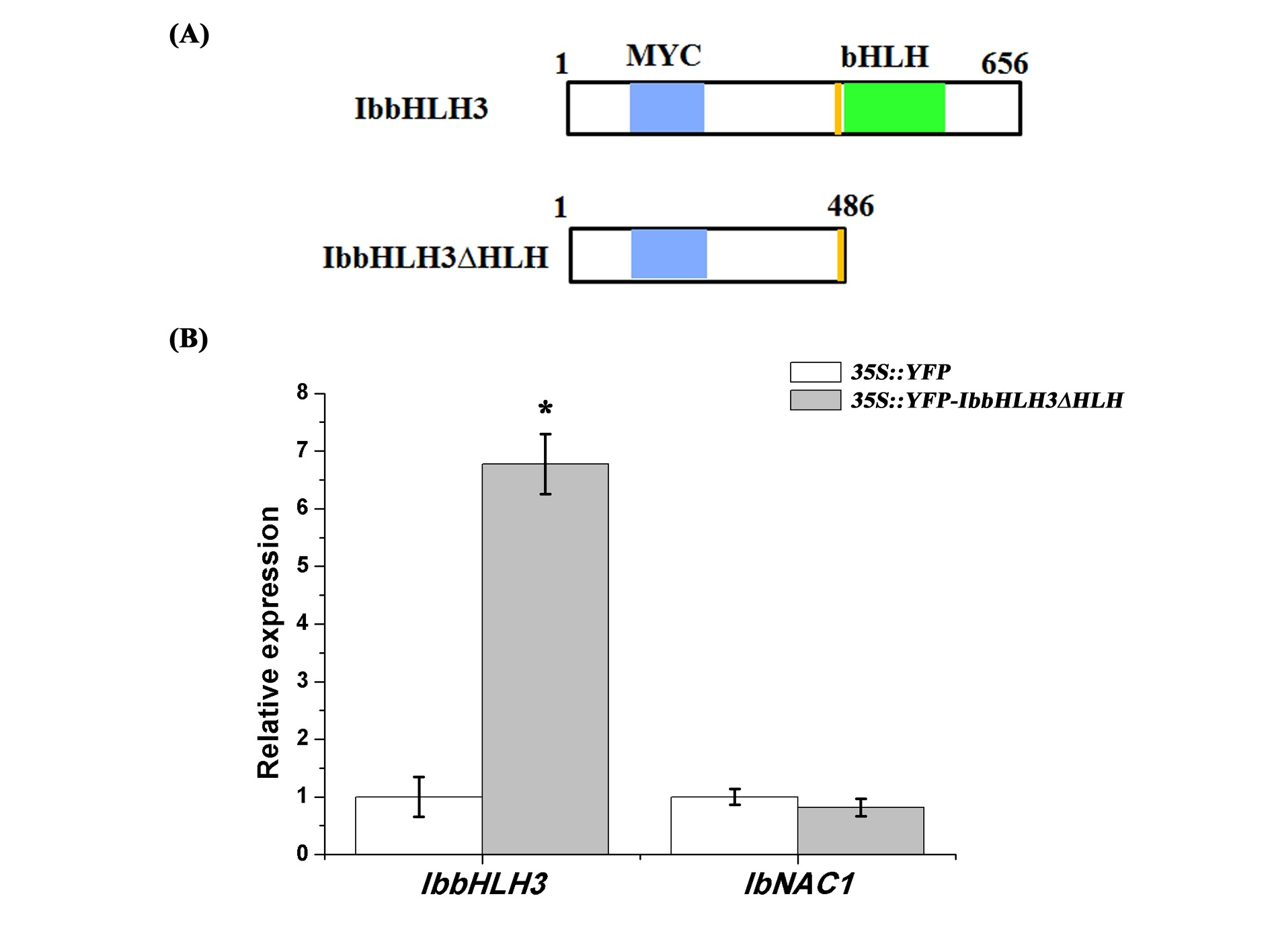

Supplement: S8 Fig — (A) A schematic representation of HLH domain in IbbHLH3. (B) The expression of IbNAC1 and IbbHLH3 in sweet potato overexpressing IbbHLH3ΔHLH. 35S::YFP-IbbHLH3ΔHLH or 35S::YFP was transiently expressed in sweet potato leaves by particle bombardment. After bombardment for 3 days, the expression of IbNAC1 and IbbHLH3 were monitored by qRT-PCR. Error bars indicate SDs (n = 5). Asterisks represent significant difference from YFP (Student’s t-test; P<0.05). (TIF) [file pgen.1006397.s009.tif]

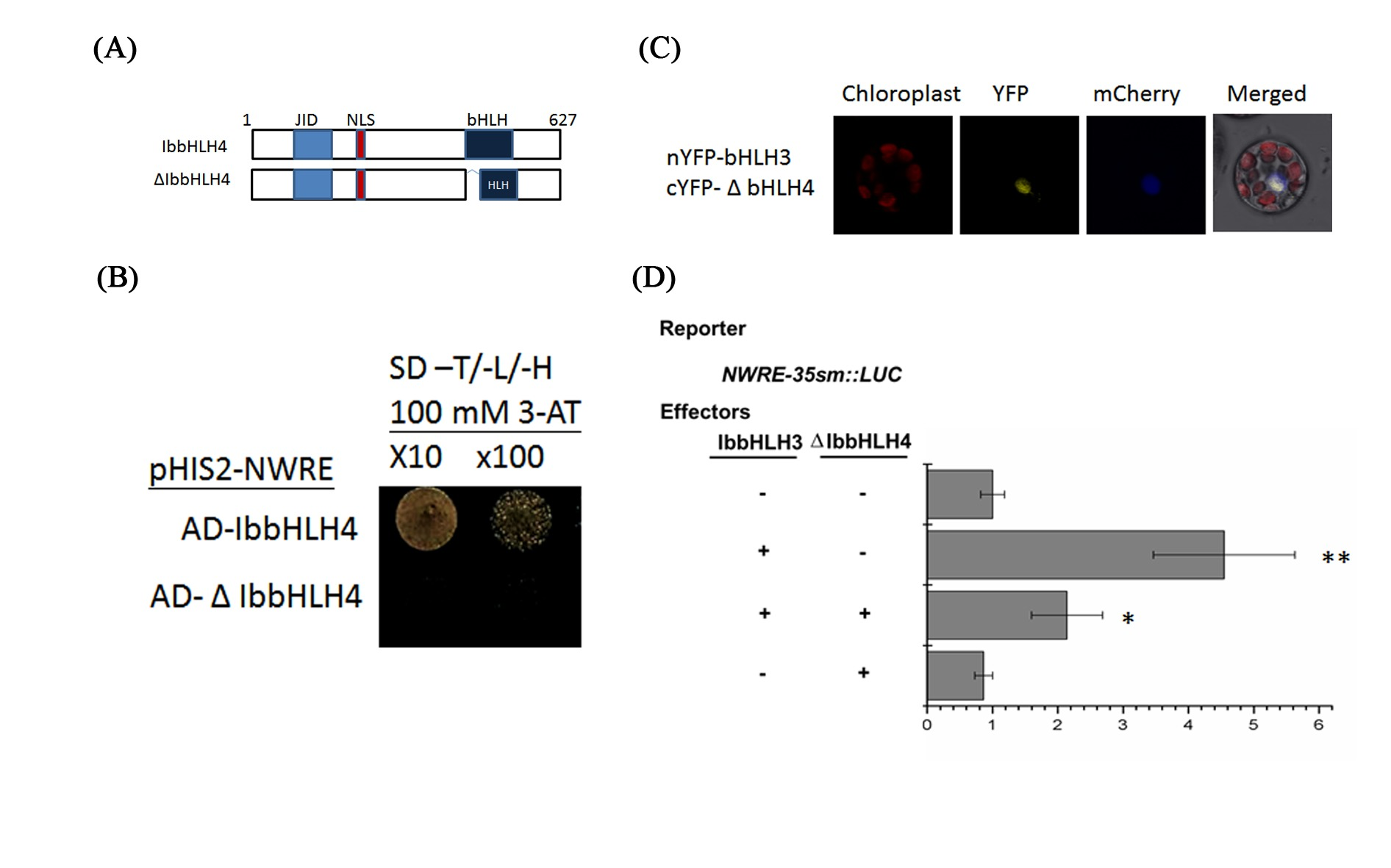

Supplement: S9 Fig — (A) A schematic model of the truncated DNA binding domain of IbbHLH4 (ΔIbbHLH4). (B) NWRE binding ability of truncated IbbHLH4. ΔIbbHLH4 was cloned into the yeast expression vector pGADT7, forming AD-ΔIbbHLH4. pHIS2-NWRE was used as bait to examine the binding ability of ΔIbbHLH4. (C) BiFC assay. ΔIbbHLH4 was fused to cYFP in the N-terminal region, and the interaction with IbbHLH3 analyzed in Arabidopsis protoplasts. (D) IbbHLH3 competing assay of ΔIbbHLH4 in regulation of the NWRE activity. Error bars indicate SDs (n = 10). Asterisks represent significant difference from reporter alone (*, P<0.05; **, P<0.01). (TIF) [file pgen.1006397.s010.tif]

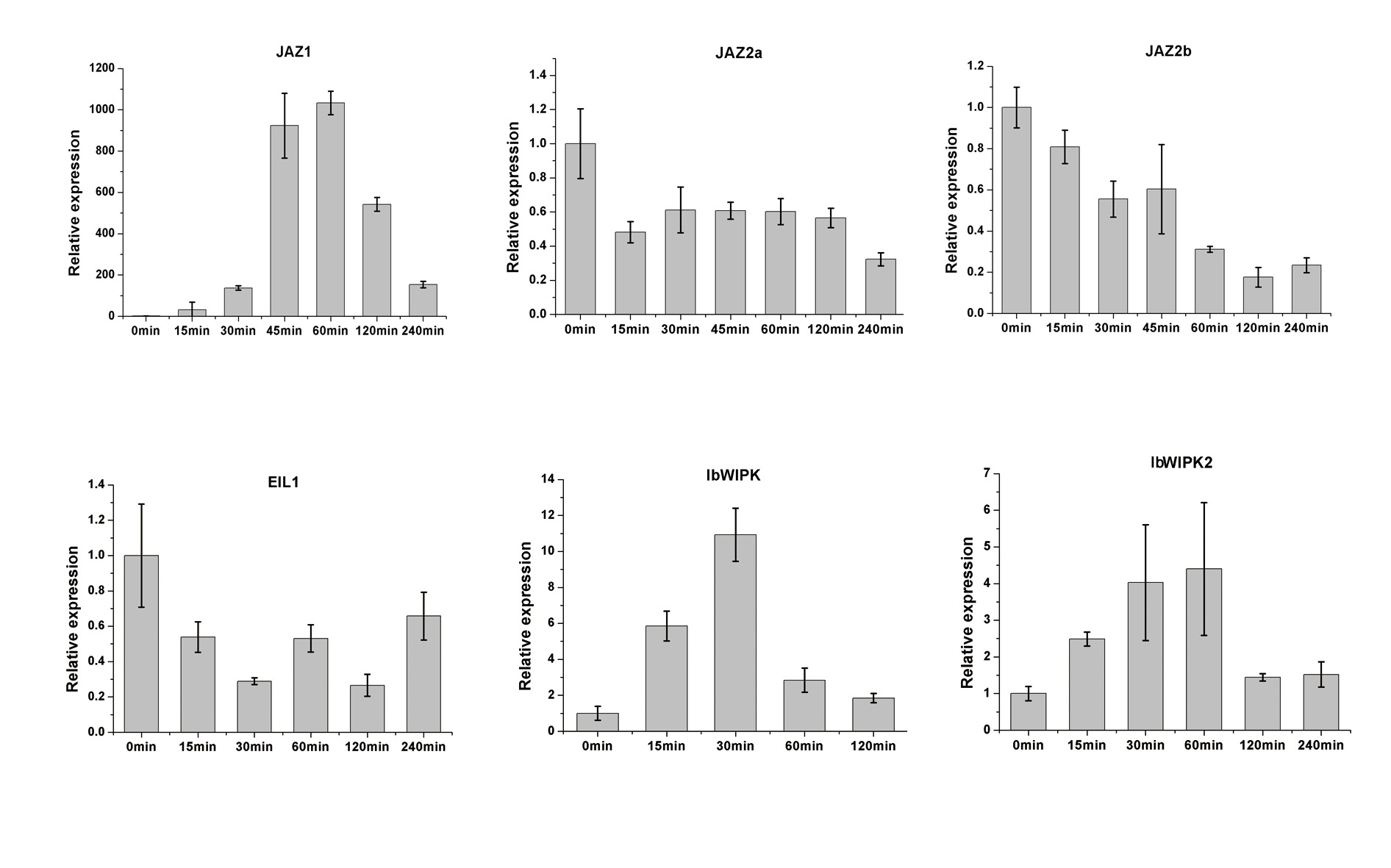

Supplement: S10 Fig — The expression of putative IbbHLH3-binding factors, including JAZ1, JAZ2a, JAZ2b, EIL1, IbWIPK1, and IbWIPK2, was monitored by qRT-PCR under a wounding time-course analysis. Error bars indicate SDs from four independent replicates. (TIF) [file pgen.1006397.s011.tif]

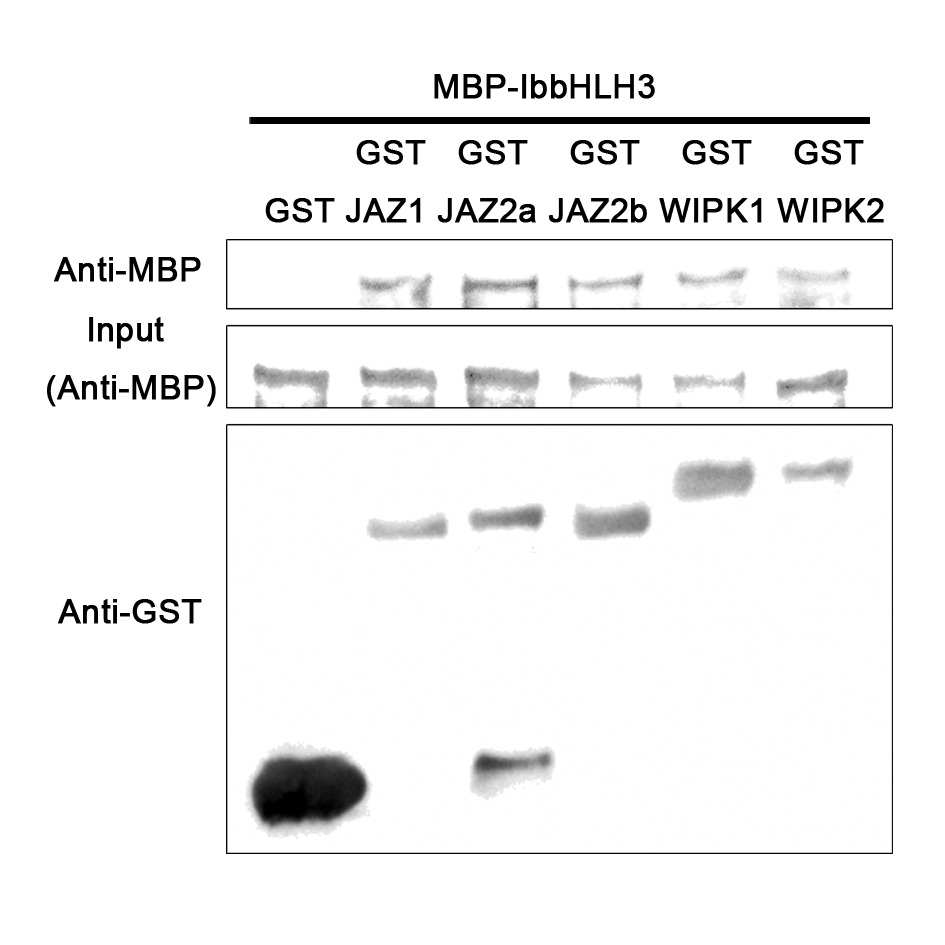

Supplement: S11 Fig — The purified MBP-IbbHLH3 protein were incubated with the GST-fusion proteins (GST-JAZ1, GST-JAZ2a, GST-JAZ2b, GST-IbWIPK1 and GST-IbWIPK2), respectively. Bound proteins were precipitated with the anti-GST agarose and further analyzed by western blot using anti-GST antibody and anti-MBP antibody. The input lane represents the protein level of IbbHLH3 before reaction. (TIF) [file pgen.1006397.s012.tif]

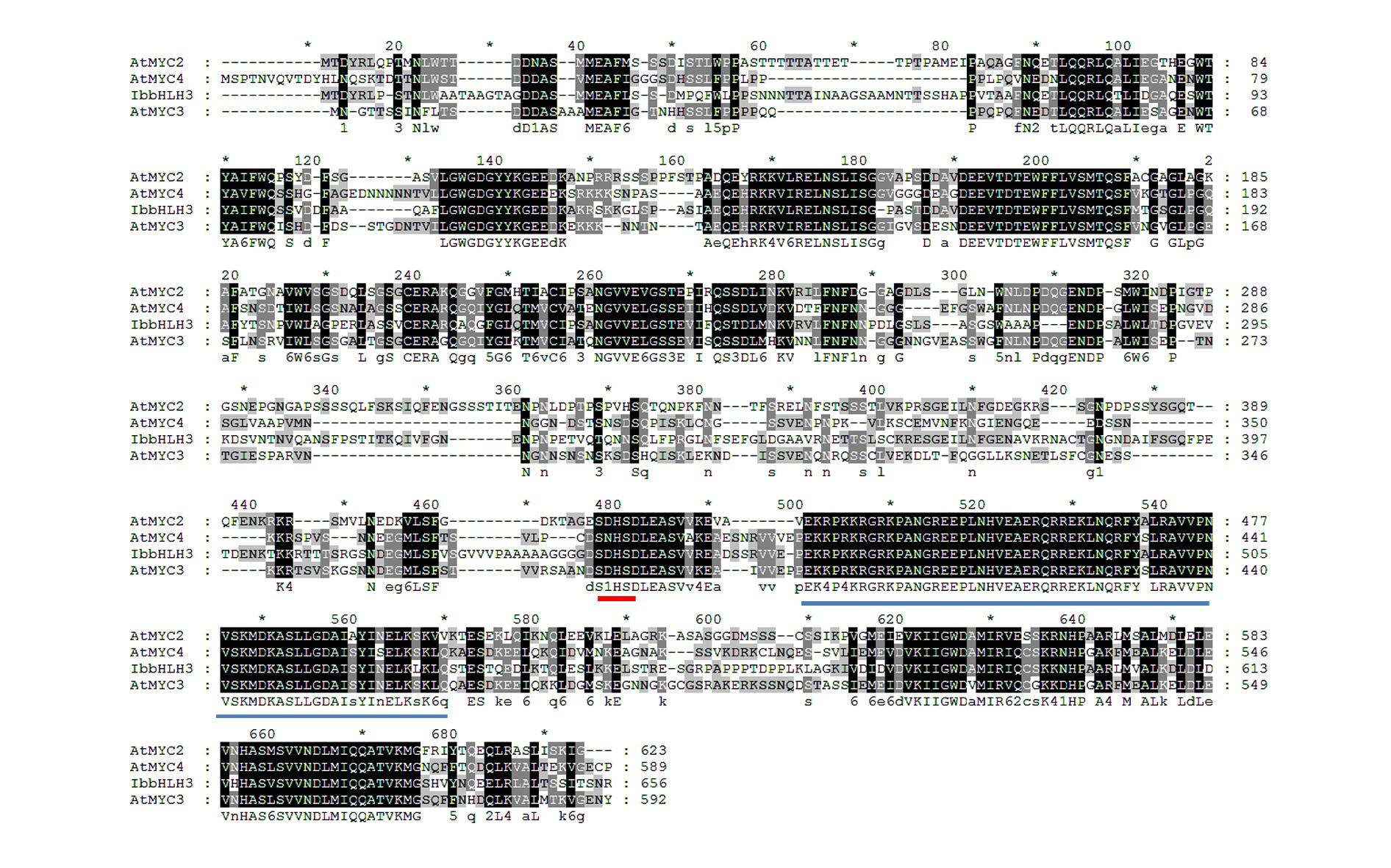

Supplement: S12 Fig — The amino sequence alignment of bHLH group IIIe transcription factors, including AtMYC2/3/4 and IbbHLH3, was analyzed by GeneDoc software. The HLH-domain (blue line) was conserved in bHLH-IIIe transcription factors. The putative phosphorylation site in IbbHLH3 was labeled as red underline. (TIF) [file pgen.1006397.s013.tif]

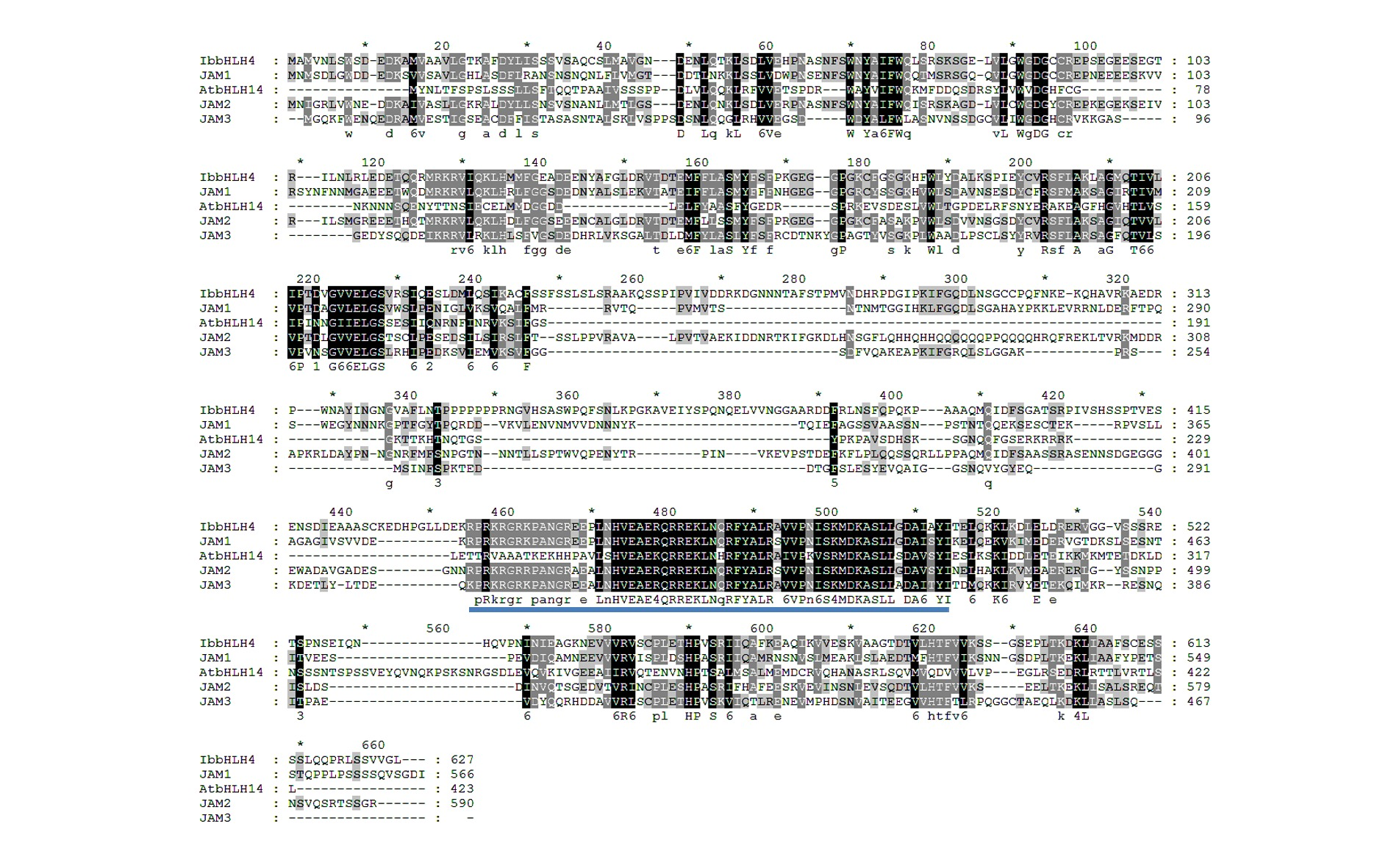

Supplement: S13 Fig — The amino sequence alignment of bHLH group IIId transcription factors, including AtJAM1/2/3 and IbbHLH4, was analyzed by GeneDoc software. The HLH-domain (blue line) was conserved in bHLH-IIId transcription factors, expect for AtbHLH14. (TIF) [file pgen.1006397.s014.tif]
